# Spatiotemporal Computations of an Excitable and Plastic Brain: Neuronal Plasticity Leads to Noise-Robust and Noise-Constructive Computations

**DOI:** 10.1371/journal.pcbi.1003512

**Published:** 2014-03-20

**Authors:** Hazem Toutounji, Gordon Pipa

**Affiliations:** Institute of Cognitive Science, University of Osnabrück, Osnabrück, Lower Saxony, Germany; Indiana University, United States of America

## Abstract

It is a long-established fact that neuronal plasticity occupies the central role in generating neural function and computation. Nevertheless, no unifying account exists of how neurons in a recurrent cortical network learn to compute on temporally and spatially extended stimuli. However, these stimuli constitute the norm, rather than the exception, of the brain's input. Here, we introduce a geometric theory of learning spatiotemporal computations through neuronal plasticity. To that end, we rigorously formulate the problem of neural representations as a relation in space between stimulus-induced neural activity and the asymptotic dynamics of excitable cortical networks. Backed up by computer simulations and numerical analysis, we show that two canonical and widely spread forms of neuronal plasticity, that is, spike-timing-dependent synaptic plasticity and intrinsic plasticity, are both necessary for creating neural representations, such that these computations become realizable. Interestingly, the effects of these forms of plasticity on the emerging neural code relate to properties necessary for both combating and utilizing noise. The neural dynamics also exhibits features of the most likely stimulus in the network's spontaneous activity. These properties of the spatiotemporal neural code resulting from plasticity, having their grounding in nature, further consolidate the biological relevance of our findings.

## Author Summary

The world is not perceived as a chain of segmented sensory still lifes. Instead, it appears that the brain is capable of integrating the temporal dependencies of the incoming sensory stream with the spatial aspects of that input. It then transfers the resulting whole in a useful manner, in order to reach a coherent and causally sound image of our physical surroundings, and to act within it. These spatiotemporal computations are made possible through a cluster of local and coexisting adaptation mechanisms known collectively as neuronal plasticity. While this role is widely known and supported by experimental evidence, no unifying theory of how the brain, through the interaction of plasticity mechanisms, gets to represent spatiotemporal computations in its spatiotemporal activity. In this paper, we aim at such a theory. We develop a rigorous mathematical formalism of spatiotemporal representations within the input-driven dynamics of cortical networks. We demonstrate that the interaction of two of the most common plasticity mechanisms, intrinsic and synaptic plasticity, leads to representations that allow for spatiotemporal computations. We also show that these representations are structured to tolerate noise and to even benefit from it.

## Introduction

Neuronal plasticity, both homeostatic and synaptic, is the central ingredient for the generation and adaptation of neural function and computation [1]. However, it remains mostly unclear how neurons in recurrent neural networks utilize neuronal plasticity to self-organize and to learn computing on temporally and spatially extended stimuli [2]–[4].

A full grasp of the principles of self-organization by plasticity in recurrent neural networks is *jointly* hampered by the diversity of existing neuronal plasticity mechanisms [5]–[7] and the limited understanding of their functions and cooperations, by the *emergent* nature of computation in recurrent systems, in the sense that computation is a collective phenomenon of the system as a whole and cannot be fully understood from the contribution of individual neurons [8], [9], and by the fact that neural systems are subject to noise [10]–[13]. In this paper, we simultaneously address these issues by studying the basic principles of self-organization in *recurrent* networks that arise from the interaction of synaptic and homeostatic intrinsic *plasticity*, and given that the network is subject to *noise*. To this end, we use numerical methods to explore the dynamics of nonautonomous, i.e. stimulus-driven, and plastic recurrent networks, and we provide a mathematical formalization for attaining a rigorously sound perspective (see Methods).

Incorporating synaptic plasticity with homeostasis goes back to Bienenstock, Cooper, and Monro's groundbreaking work known as the *BCM theory* [14]. Through rigorous mathematical analysis, the BCM theory predicted the necessity of a certain form of a sliding threshold, i.e. a homeostatic adjustment of neuronal excitability, for stabilizing the plastic afferent weights of a single neuron. Empirical findings supported the hypothesis of adjustable excitability and showed that it manifests through changes of neuronal properties at the soma [6], [7]. While the BCM theory suggests homeostasis as a stabilization mechanism of synaptic weights with no direct influence on the neuron's encoding properties, Triesch proposed a homeostatic ***i*** *ntrinsic* ***p*** *lasticity* (IP) mechanism that increases the neuron's encoding capacity and cooperates with ***s*** *ynaptic* ***p*** *lasticity* (SP) to discover nonlinear independent features of the neuron's inputs [15].

These investigations, among others [16], [17], are very insightful in pinpointing how synaptic and homeostatic plasticity interact in *single neurons*. In addition, *feedforward* neural networks greatly simplify the analysis and understanding of self-organization and computation based on neuronal plasticity. For such architectures, both single plasticity rules, as well as combinations of different plasticity mechanisms, had been linked to neural computation, such as the formation of receptive fields [14], the related identification of statistically-independent components [15], [17], [18], and predictive coding [19]. However, it is important to note that neurons are embedded within large and highly recurrent networks [20]–[23], and that an efficient use of neuronal resources entails distributed encoding schemes [8], [9]. In addition, besides the spatial features of the world, its temporal structure should also be captured by the neural code [4], [24]–[26].

Our understanding of neural information processing would greatly improve by extending the principles of self-organization to recurrent neural circuits, since the latter constitute the basic computational units in the cortex [22]. Lazar et al. were the first to study the emergence of computation from the interaction of different forms of plasticity on recurrent neural networks [27], [28]. This study builds on their findings. However, we *do not* restrict the definition of computation to linear classifiers of the ***r*** *eservoir* ***c*** *omputing* (RC) paradigm [3], [29], [30]. In addition to training linear classifiers for measuring the *computational performance*, we identified the necessity of analyzing the response of the recurrent neural network itself as an *input-driven dynamical system* [31], [32], and of concurrently viewing the network as a *communication channel* by taking an *information-theoretical* perspective [33]. Combining these tools enables us to understand how information is encoded in recurrent systems, how such encoding is developing from self-organization, and how noise is effecting both.

Analyzing the dynamics of a *large* and, most importantly, *input-driven* neural system shaped by biologically-relevant *plasticity* is a hard task due to several methodological constraints. First, most analysis tools from *dynamical systems theory* are confined to small dynamical systems with very few degrees of freedom [34]. Exceptions are studies that circumvent this limitation by focusing on the low-dimensional collective dynamics of neural networks, e.g., [35], or studies that probe the high-dimensional phase space of the neural network, such as the classic example of Hopfield Networks [36]. Other instances of high-dimensional dynamical systems include ring networks and their coexisting periodic attractors [37], stable heteroclinic orbits [38], [39], unstable periodic attractors [40], and others [41]–[43].

The second and most important methodological constraint is that the use of standard dynamical systems theory is inappropriate, since it deals with autonomous systems only, i.e. systems with no explicit dependence on time. In reality, however, neural networks are subject to a flux of ever changing stimulation that renders them nonautonomous. A theory of *nonautonomous dynamical systems* is only recently taking shape as a branch of applied mathematics [31], [32]. The fields of neural computation and computational biology are constantly contributing to the theory with concepts such as meta-transients and attractor morphing [37], [44], *γ*-systems [45], and the nonautonomous dynamics of echo state networks [46].

A simple intuition of the difference between nonautonomous and autonomous systems can be stated as follows. Attractors of an autonomous dynamical system are defined by the system alone, and are therefore fixed. In contrast, attractors of a nonautonomous system are jointly defined by the dynamical system and its input. As the input changes, so does the attractor landscape of the system. This highlights the fact that studying computations in a driven system using the methods of autonomous dynamical systems is insufficient, since the input-induced changes of the system, i.e. changes of its attractor landscape, are ignored in that case.

The third constraint is that the complexity of the dynamics increases due to the neural system's adaptability. The presence of plasticity imposes restrictions on the dynamics a network can exhibit, thus keeping the network dynamics in a regime that can support complex computations. To the best of our knowledge, no attempt prior to this work has been taken to combine high-dimensionality and nonautonomy with the consequences of plasticity on dynamics. We demonstrate that plasticity *sculptures* the stimulus-specific dynamic landscapes, and by that, serves in improving representation of the provided input. Moreover, neuronal plasticity can adapt and learn stimulus-induced sequences of such stimulus-specific landscapes. We thereby show that neuronal plasticity improves spatiotemporal computations.

Given the above, we highlight and explain that spatiotemporal computations require two basic ingredients: a homeostatic mechanism that regulates neuronal activity, and synaptic learning that adapts the network's recurrent connectivity to the stimulus. We show that combining both types leads to a system that: first, learns the temporal structure of the input and carries out nonlinear computations, second, is noise tolerant, and third, even benefits from the presence of noise that sets the system to an input-sensitive dynamic regime.

The paper is structured as follows. We first characterize the effects of self-organized adaptation that is based on synaptic and homeostatic intrinsic plasticity and their combination. For that, we use tasks where both random and temporally-structured inputs are reconstructed and predicted, as well as a task where nonlinear computations are performed. We estimate the network's self-information capacity (its *entropy*), and its input-information capacity (the *mutual information* between the input and the network). We then interlude to qualitatively analyze the resulting dynamics of plastic changes based on the theory of nonautonomous dynamical systems. We explain the superior computation of conjoining synaptic and intrinsic plasticity based on both the informational and dynamical analyses. Building upon that, we study network noise, and demonstrate how noise is combated and exploited through the interaction of synaptic and intrinsic plasticity.

## Results

**Figure 1:**
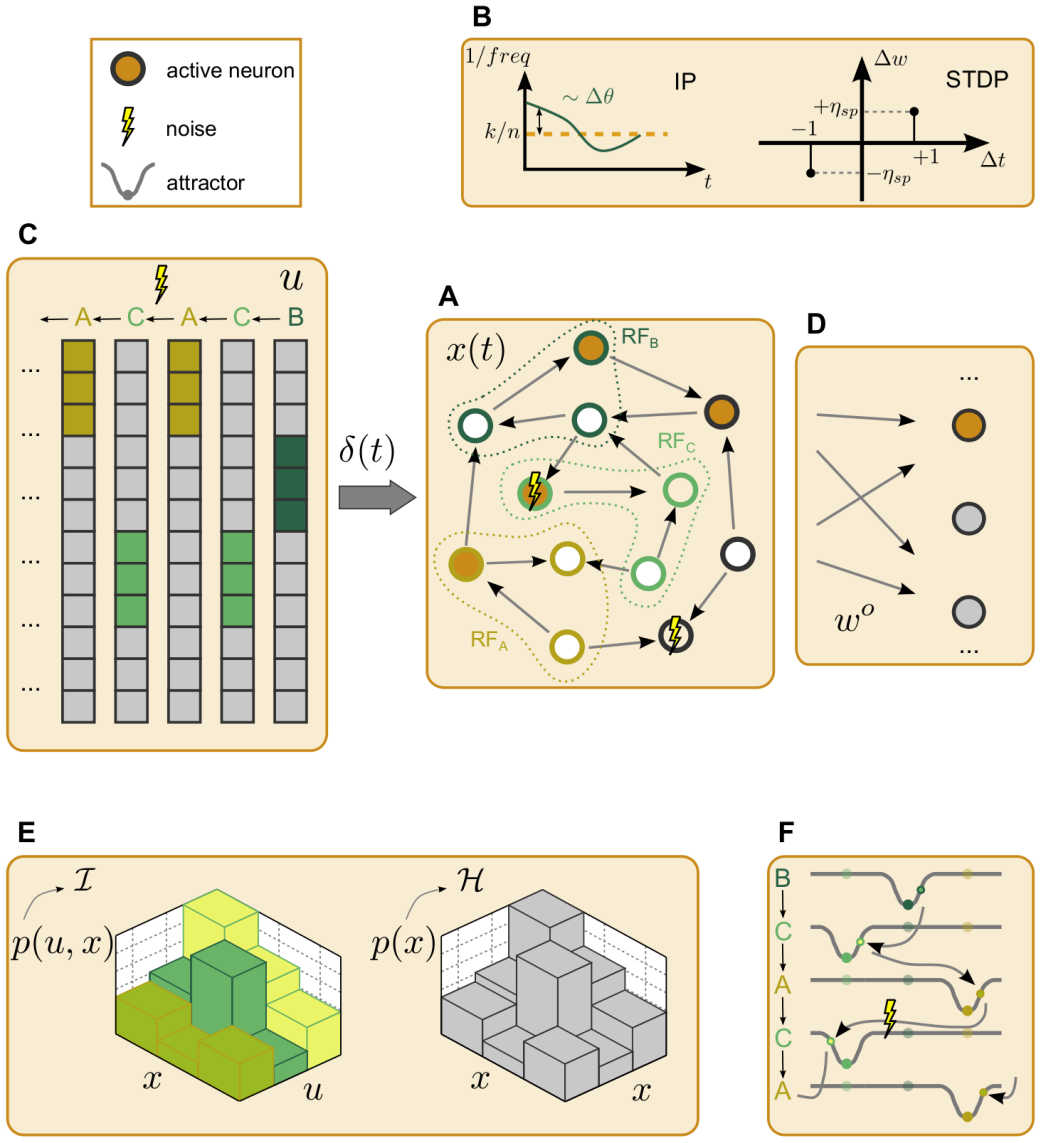
Overview of the recurrent network model and the methods for analyzing its computational capabilities. (A) An exemplary recurrent neural network of 12 neurons. The network state 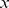 has a 4-Winner-Take-All (4WTA) nonlinear dynamics, where the 4 neurons with the highest membrane potential fire and the rest are silent. The membrane potential is the sum of the recurrent afferents and the external drive 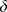. It is also depolarized (hyperpolarized) with decreasing (increasing) excitability threshold 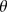. The recurrent network can also be subject to *noise*, while reserving the 4WTA dynamics: when a neuron fails to spike due to noise, another fires instead. (B) The recurrent network is adapted by two plasticity mechanisms. The excitability threshold 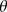 is modulated by ***i*** *ntrinsic* ***p*** *lasticity* (IP), the recurrent afferents 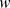 by ***s*** *pike-* ***t*** *iming-* ***d*** *ependent synaptic* ***p*** *lasticity* (STDP). (C) The external drive 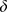 consists of discrete symbols that follow a certain stochastic dynamics, and each projects to a corresponding ***r*** *eceptive* ***f*** *ield* (RF). The exemplary drive is a 3-symbols Markov chain 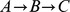 that allows a probability for *noisy* transitions, i.e. 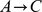. (D) Linear functions of the network state 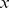 parametrized by output weights 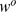 fitted to (possibly nonlinear) target functions of sequences of the external drive. (E) Nonlinear information-theoretic quantities are measured: network state entropy 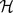 and the mutual information 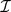 of the network state 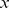 and input sequence 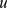. (F) Analysis of the appearance and disappearance of attractors due to the external drive within the network as an input-driven dynamical system.

In this section, we guide the reader through the following topics. We start by elucidating the computational power gained through the combination of synaptic and homeostatic plasticity mechanisms on recurrent neural networks of the *k-Winner-Take-All* (kWTA) type. We investigate the role of these plasticity forms in shaping the neural code through their effects on the informational and dynamical landscapes of the network. We conclude by illustrating how synaptically and homeostatically organized recurrent networks both benefit from noise and tolerate its presence. Figure 1 schematically illustrates the network model, the plasticity rules, and the formal probes we used to evaluate and describe the resulting computational properties. More details are available in the Methods section.

### Computational Power

The interaction of different forms of plasticity produces a rather complex emergent behavior that cannot be explained trivially by the individual operation of each. We therefore start with exploring the effects induced by the combination of ***s*** *pike-* ***t*** *iming-* ***d*** *ependent synaptic* ***p*** *lasticity* (STDP) and ***i*** *ntrinsic* ***p*** *lasticity* (IP). We compare the computational performance of recurrent networks trained either with both synaptic and intrinsic plasticity (SIP-RNs), with synaptic plasticity alone (SP-RNs), or with intrinsic plasticity alone (IP-RNs), in addition to nonplastic recurrent networks, where the synaptic efficacies and firing thresholds are random.

Following the *plasticity phase*, a network is *reset* to random initial conditions and the *training phase* starts. Output weights from the recurrent network to linear readouts are computed with linear regression so that the readouts activity is the optimal linear classifier of a target signal. The target signal depends on the computational task. That is followed by the *testing phase*, at which performance is computed. Performance is measured by the percentage of correctly matched readout activity to the target signal.

Naturally, during simulation, the recurrent network is excited by a task-dependent external drive. The battery of tasks we deployed was designed to *abstract* a certain aspect of the spatiotemporal computations faced by biological brains, i.e. recalling past stimuli, predicting future ones, and nonlinearly transforming them. The memory task RAND x 4, the prediction task Markov-85, and the nonlinear task Parity-3, as well as the plasticity models and simulation conditions, are detailed in the Methods section.

**Figure 2:**
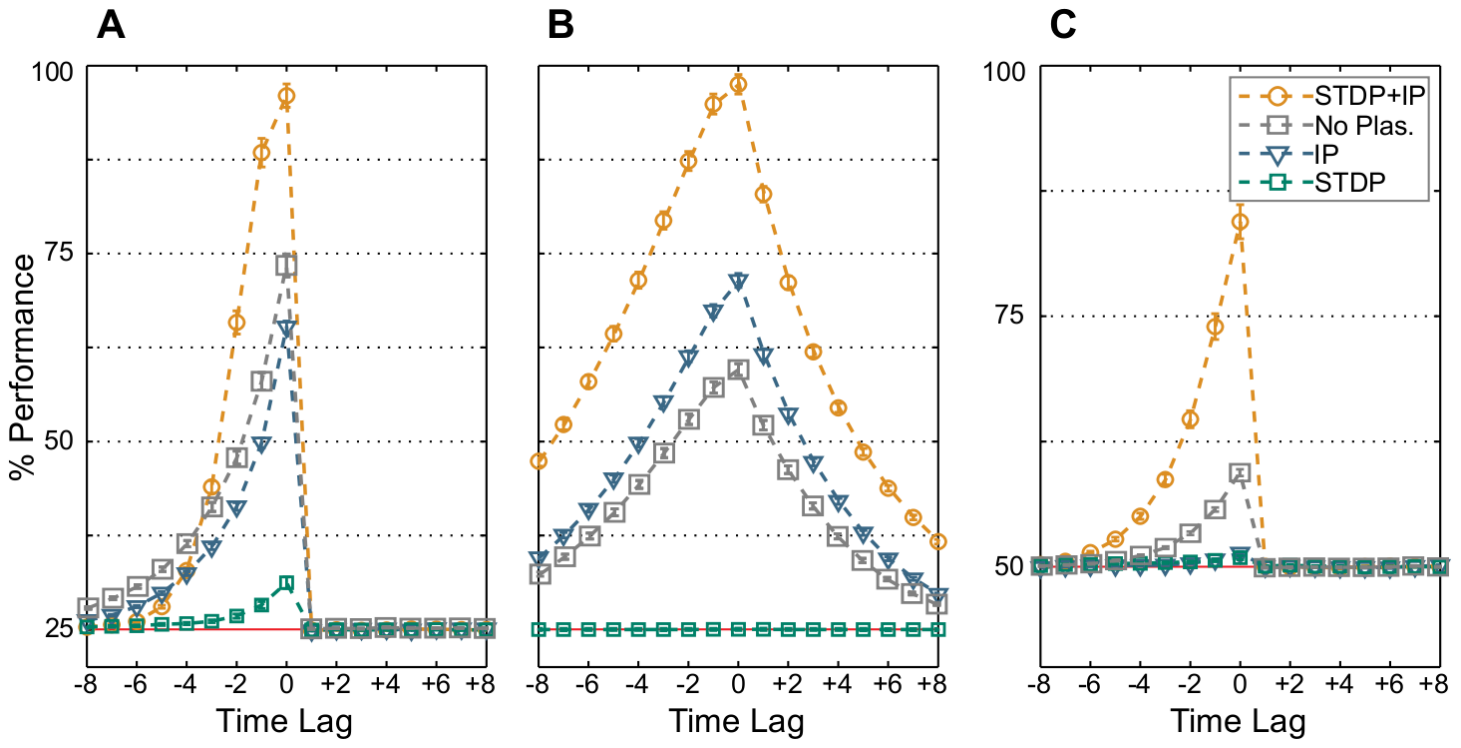
Average classification performance. 100 networks are trained by STDP and IP simultaneously (orange), IP alone (blue), STDP alone (green), or are nonplastic (gray). Optimal linear classifiers are then trained to perform (A) the memory task RAND x 4, (B) the prediction task Markov-85, and (C) the nonlinear task Parity-3. Nonplastic networks have their weights trained by STDP and then randomly shuffled, so that they have the same weight and threshold distributions as SP-RNs. However, due to the shuffling, their weight matrices carry no structure. Error bars indicate standard error of the mean. The red line marks chance level. The *x*-axis shows the input time-lag. Negative time-lags indicate the past, and positive ones, the future.

Figure 2 shows that SIP-RNs significantly outperform both IP-RNs and SP-RNs in all tasks. Inputs from 3 time steps in the past are successfully retained far beyond chance level in the memory task RAND x 4 (Figure 2A). Understandably, performance drops to chance level for future stimuli (positive time-lags), since input symbols are equiprobable and their temporal succession carries no structure. Such is the case for the nonlinear task (Figure 2C). It is worth noting that solving the nonlinear task Parity-3 requires recalling three successive stimuli, which adds to the computational load. The recurrent network, through learning the temporally-structured input of the task Markov-85, boosts the readouts' ability to reconstruct past symbols in comparison to the structureless memory task RAND x 4. It also allows for the prediction of future stimuli far beyond chance (Figure 2B).

**Figure 3:**
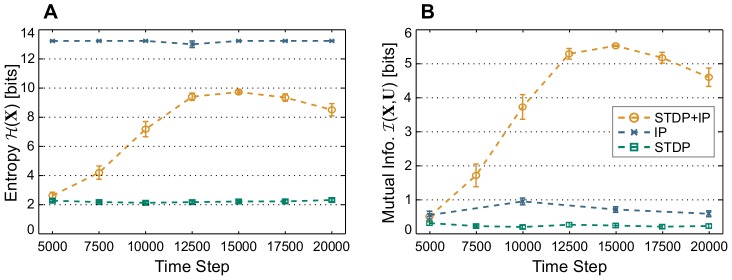
Network state entropy and the mutual information with input. (A) Network state entropy 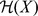 and (B) the mutual information with the three most recent RAND x 4 inputs 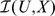 as they develop through the plasticity phase for SP-RNs (green), IP-RNs (blue), and SIP-RNs (orange). Mutual information for IP-RNs is estimated from 500000 time steps, and is averaged over 5 networks only. Other values are averaged over 50 networks and estimated from 100000 samples for each network. Error bars indicate standard error of the mean.

STDP alone fails to provide the recurrent network with means to encode necessary information. This leads to SP-RNs performing at almost chance level in all tasks. Intrinsic plasticity, on the other hand, endues recurrent networks with an intermediate ability to sustain past inputs (Figure 2A). IP-RNs also seem to learn the temporal structure of the input, as optimal linear classifiers are capable of predicting future stimuli (Figure 2B). Intrinsic plasticity is, however, insufficient for nonlinear computations, as IP-RNs barely perform above chance in the nonlinear parity task.

We also compare the performance of nonplastic kWTA networks with similar weight and threshold distributions as SP-RNs (shown in gray in Figure 2). They perform better than IP-RNs on the memory and nonlinear tasks, and worse on the prediction task. In all tasks, these nonplastic networks perform worse than SIP-RNs. We also show in Text S1 that nonplastic networks with comparable weight and threshold distributions as SIP-RNs also perform significantly lower than plastic networks. These results supply the evidence that the presence of plasticity enhances the computational power of recurrent neural networks (see Text S1 for a discussion on heuristics for finding comparable random networks). No further analysis is carried out on these nonplastic networks, since the aim of this paper is to discern the effects of synaptic and intrinsic plasticity on spatiotemporal computations.

### Neural Code

Explaining the superiority of networks modified by deploying both STDP and IP starts from isolating the individual role of each plasticity mechanism in defining the spatiotemporal neural code. In that regard, a well-informed intuition is that STDP learns the basic structure of the input as the connectivity resulting from STDP reflects the input sequence transitions. IP, on the other hand, increases the neural bandwidth by introducing redundancy to the code, as IP leads to the longest periodic cycles in the spontaneous activity of kWTA networks (See Figure 8 and Figure 4A in [27]).

**Figure 4:**
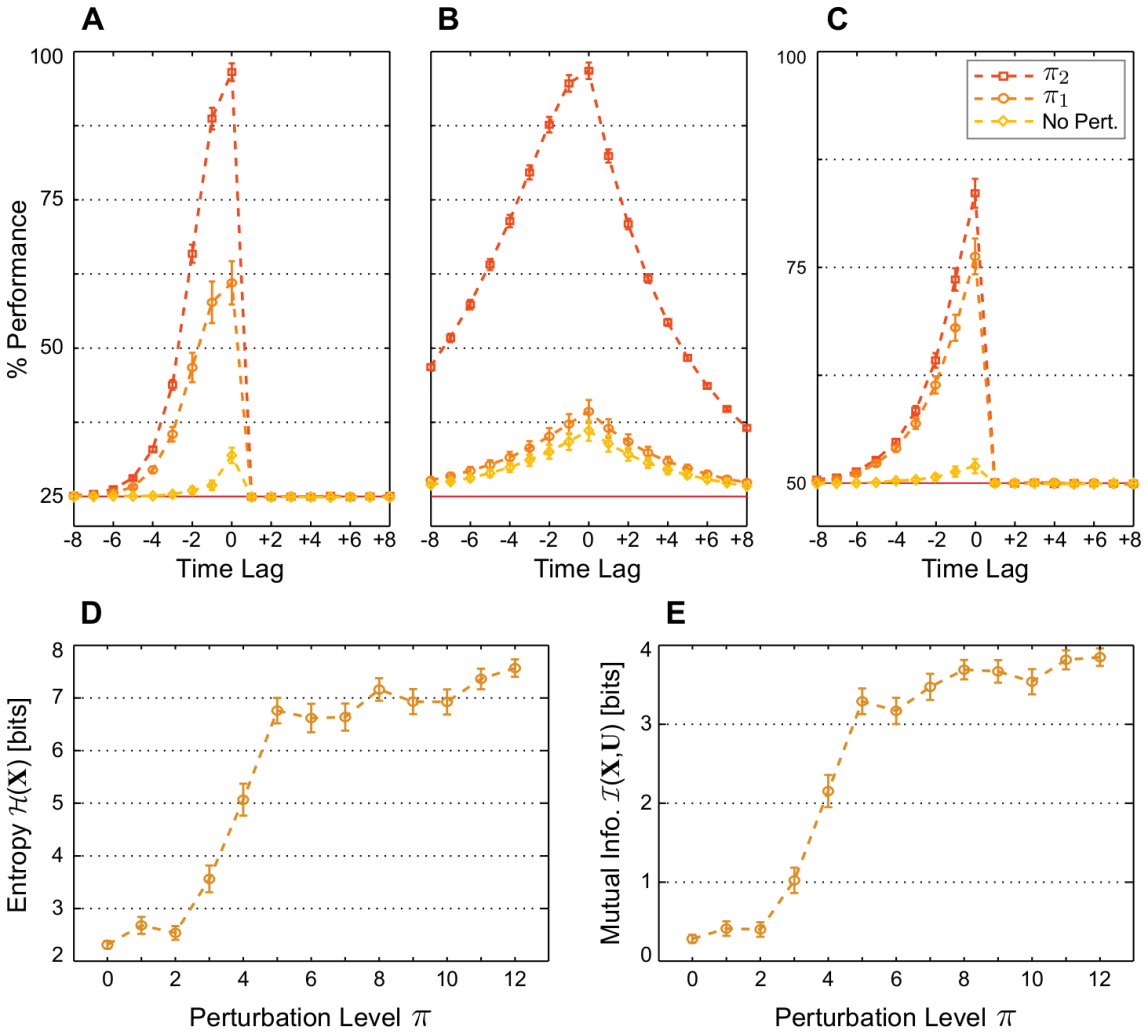
Post-plasticity perturbation. 100 networks are trained by STDP and IP simultaneously on (A) the memory task RAND x 4, (B) the prediction task Markov-85, and (C) the nonlinear task Parity-3 with increasing perturbation level: 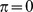 (yellow), 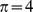 (orange), and 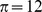 (red). Error bars indicate standard error of the mean. The red line marks chance level. The 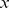-axis shows the input time-lag. Negative time-lags indicate the past, and positive ones, the future. (D) Network state entropy 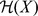 and (E) the mutual information with the three most recent RAND x 4 inputs 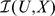 at the end of the plasticity phase for different perturbation levels. Values are averaged over 50 networks and estimated from 5000 samples for each network. Error bars indicate standard error of the mean.

The spatiotemporal neural code, or the *neural code* for short, can be characterized by both the *absolute capacity* of the network activity to store information and by how network activity *encodes* the spatially and temporally extended network input. *Entropy* of the network activity measures its absolute capacity, i.e. the repertoire of network states that the network can actually visit and potentially assign to some input sequence. The assignment of a network state to an input sequence means that this particular network state *encodes* or *represents* that input sequence. *Mutual information* between network input sequences and network states quantifies the extent of how successful this assignment is. Not every visited network state needs be assigned an input sequence. A redundant code is reflected by input sequences being represented by multiple network states. Also, a network state might fail to encode an input, thus reflecting uninformative noise states.

We investigate the neural code characteristics of kWTA networks by estimating both the entropy of the network state and the mutual information between network input sequences and network states. We drive the network by RAND x 4 input, and for computational tractability, we limit the estimation of mutual information to three-step inputs. An optimal encoder of this input sequence will then be a network with 6 bits of mutual information. The information-theoretical quantities are computed at intervals of the plasticity phase under the three plasticity conditions. At these intervals, the plastic variables are fixed and the driven network is reinitialized and run for a sufficient number of steps, and passed along with the input to the entropy and mutual information estimators. More details on how these measurements are carried out are found in the Methods section.

Figure 3 shows how these measures develop through the plasticity phase (For a discussion on the effects of longer plasticity exposure, see Text S2). SP-RNs' entropy remains constant at 2 bits. This means that SP-RNs visit only 4 network states (green in Figure 3A). However, these network states encode no information of the input sequence, as mutual information remains practically zero (green in Figure 3B). We call this 2 bits input-insensitive code the *minimal code*, as it captures no more than a single possible succession of the 4 inputs. This effect is the result of the interaction between the machination of STDP and the initial firing thresholds and weights configuration. Transitions, such as 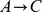 in the input space, are to be stored in some of the synapses that connect neurons in the receptive field of 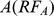 with those in the receptive field of 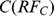. At each time step, one transition, such as 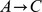, could be easier to reinforce with the causal (potentiating) side of STDP for 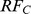 neurons having little higher excitability (internal drive plus their own firing threshold). Without IP to tune down this excitability and with further contribution from the recurrency of the network, a positive feedback loop is generated, and this transition becomes more and more potentiated at the expense of others. This transition then becomes independent of the actual drive the network is receiving: the network becomes input-insensitive.

On the other side of the entropy spectrum, we find IP-RNs. Through IP's constant adjustment of the neuronal excitability, many neurons contribute to the neural code and IP-RNs visit a large number of states. Entropy and the network state bandwidth are the highest (blue in Figure 3A). One may view IP's effect as an introduction of *intrinsic deterministic noise* to the network activity. The increase in bandwidth of the network activity raises the odds for the random weights of an IP-RN to store an input sequence. In fact, many network states encode the same input sequence, resulting in a redundant code. However, without a synaptic reinforcement of representations, many states are visited due to the internal dynamics of the network, and not due to the external drive. These states remain uninformative and input sequences not successfully encoded: the mutual information (blue in Figure 3B), and hence the classification performance, are low.

The development of the neural code for SIP-RNs follows, however, a more interesting path. At the beginning, STDP has the upper hand and a 2 bits minimal code is generated. Through providing intrinsic deterministic noise, IP enriches the neural code by increasing redundancy and entropy (orange in Figure 3A). At the same time, STDP incrementally associates different network states to different input sequences by adjusting the synaptic weights as seen from the increase of mutual information (orange in Figure 3B). Then together, synaptic and homeostatic plasticity cooperate to create a code that is both *redundant* and *input-specific*. These properties are crucial for noise-robustness, as will be shown later in this text.

### Post-Plasticity Perturbation

A dynamical system's behavior depends on its past activity. Therefore, testing a system requires assuming plausible initial conditions. The recurrent neural network at hand, even though it is small in comparison to a real neural circuit, has a number of possible initial conditions too large for all its initial conditions to be tested. So far, we have chosen random initial conditions for the network activity following the plasticity phase. From now on, we choose the initial conditions systematically by reinitializing the network activity depending on a *perturbation* 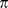. This perturbation is applied to the end state of the plasticity phase, such that the end state of the plasticity phase and the initial state of the training phase are at a distance 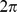 from one another. For details of how the initial conditions are selected depending on the parameter 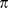, we refer the reader to the Methods section.

To discern the effect of this perturbation, we compute the performance of the trained system with the three combinations of synaptic and intrinsic plasticity. We do this both for a system that is perturbed and for a system that starts from the last state that the dynamics reaches at the end of the preceding plasticity phase. We find no difference between the two cases of initial conditions for either IP-RNs or SP-RNs. However, when the neural network is trained by both synaptic and intrinsic plasticity (SIP-RNs), we find that the perturbed networks have better performance, as is illustrated in Figure 4A–C. The high performance of SIP-RNs that results from random initial conditions, as is shown in Figure 2, is easily explainable. It stems from the fact that random initialization is merely a large perturbation, since the probability of choosing a random state from such a large set of possibilities that is at a small distance from a particular region of the state space is insignificant, compared to a state that is at a large distance. Moreover, we find that regardless of the task, larger perturbations result in higher average performance. This is also reflected in the neural code, where network state entropy and the mutual information with input correlate with higher perturbation (see Figure 4D–E).

This suggests that within the phase space of SIP-RNs there exist at least two dynamic regimes. Post-plasticity perturbation also provides the first sign of how SIP-RNs can benefit from noise, as it might put the system in the regime more suitable for computation.

### Dynamic Regimes

Optimal linear classifiers show that kWTA networks equipped with both homeostatic and synaptic plasticity are capable of creating spatiotemporal codes and performing nonlinear computation. Measuring entropy and mutual information allows for a quantification of the emerging neural code. But what are the geometric features of the neural code that allow for such computations? How do network states *represent* the spatiotemporal input in a useful way? A major part of the Methods section is devoted to developing the mathematical formalization of discrete-time nonautonomous dynamical systems. References to definitions, a proposition, and a theorem from that section are featured in the following results, as we apply these concepts to our model neural network. We view this treatment not merely as an exercise in mathematics. It allows for a rigorous description of the computational properties emerging from plasticity that are beyond the scrutiny of quantitative measures, such as linear classification performance and carried information. A consequence of these properties is also the two noise-related features we examine later.

For a formal treatment of spatiotemporal computations which result from plasticity, we need to extend the theory of nonautonomous dynamical systems to provide a notion for representations, to specify how these representations allow for computations, and to discern the effect of plasticity in enhancing these representations for the sake of computation. But first, we start by identifying the modes of operation, i.e. the dynamic regimes, the model plastic neural network has, since not all regimes might be suitable for computation.

According to Proposition 3 and Definition 6, when subject to stimulation, kWTA networks are *input-driven discrete-time dynamical systems*. For such systems, two extremes exist regarding the degree of sensitivity the system exhibits in response to its input. At one extreme, the system shows no change of response for different inputs, so that it follows its own dynamics, as if no input exists. In such a mode of operation, the system is *input-insensitive*. The other extreme is when the system's response is different for each input and initial condition. A single system can show, in principle, multiple modes of operation, depending on the initial conditions. The set of initial conditions that show a single mode of operation defines a dynamic regime and a basin of attraction.

**Figure 5:**
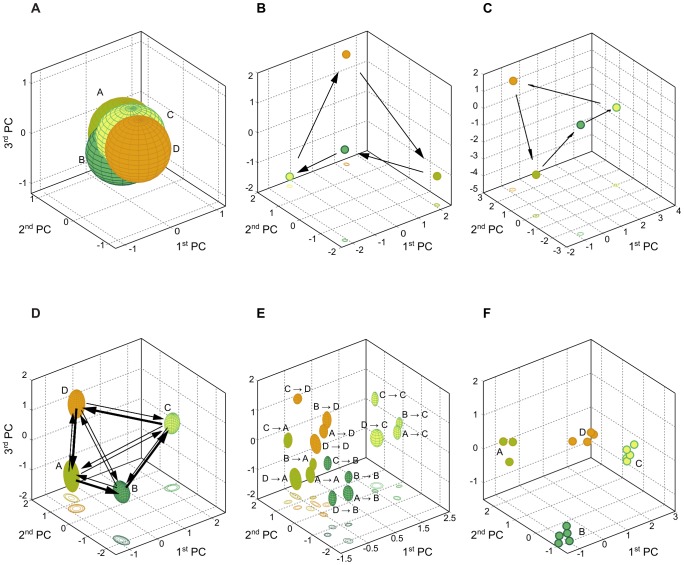
Plasticity effects on networks dynamics and input representations under the prediction task input. The three dimensions correspond to the first three principal components (PCs) of the network activity. (A) Highly-overlapping order-1 volumes of representation of an IP-RN. (B) Input-insensitive global attractor of a SP-RN that corresponds to a minimal code. (C) With no perturbed (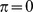), a SIP-RN dynamics also converges to an input-insensitive attractor and exhibits a minimal code. (D) Approximate visualization of order-1 volumes of representation of a SIP-RN. The approximation uses the means and the standard deviations of the corresponding coordinates of the network activity in the principal components space as the center and semi-axes lengths of ellipsoids. Arrows correspond to the transitions from one input symbol to the other. Their thickness symbolizes the probability of a transition, which reflects the Markov-85 transition probability. The collection of volumes of representation and the arrows show the perturbation set within which the nonautonomous attractor resides. (E) Order-2 volumes of representation of a SIP-RN also approximated using the mean and standard deviations of coordinates. Order-2 volumes are more exact approximations to the order-1 representations according to the volumes' inclusion property. The correspondence is clarified by using similar color coding. (F) Autonomous periodic attractors of a SIP-RN, each belonging to one of the autonomous semi-dynamical systems associated with one Markov-85 input. For clarity, no arrows are drawn between the vertexes of an attractor.

In a first step, we visualize the high-dimensional response of the system to its input. To that end, we down-project the network activity to the first three principal components, and we study the effects of STDP and IP on the network's dynamics and input representations in this reduced 3-dimensional space (Figure 5). This analysis is performed on networks with Markov-85 input which fully demonstrate the relevant properties. It is important to note that while our analysis concerns the dynamics following the plasticity phase, we are still able to infer how it unfolds during this phase from the development of the neural code (Figure 3), as we make clear later.

As suggested by the performance of SP-RNs (Figure 2) and their neural code entropy and mutual information (Figure 3), their state space is dominated by an *input-insensitive* basin of attraction and these networks behave like autonomous semi-dynamical systems (prefixing with “semi” refers to the fact that the dynamics needs not be invertible). This is confirmed by the asymptotic dynamics of SP-RNs, which is independent of the input (Figure 5B). The dynamics within this dynamic regime follows the minimal code. The minimal code manifests itself through a period-4 periodic attractor which corresponds, in the case of Markov-85 input, to the most probable transition in the input space 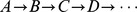. This observation confirms the fact that STDP allows the system to learn the basic structure of its input.

SIP-RNs exhibits similar dynamics at the end of the plasticity phase (Figure 5C). However, as is evident from varying the perturbation parameter for SIP-RNs (Figure 4), the set of initial conditions that constitutes this input-insensitive basin is confined by a distance relation to the neighborhood of the periodic attractor: the probability of being in this basin diminishes the further away the initial conditions are from the input-insensitive periodic attractor.

The increase of performance and the neural bandwidth of SIP-RNs for higher 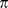 (Figure 4) shows that outside of the *input-insensitive* dynamic regime there exists a different basin of attraction. Within this basin, the network is sensitive to input, and computations are possible. The observation that 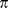 has no effect on IP-RNs and that they show intermediate performance and mutual information suggests that they are dominated by a dynamic regime with intermediate input-sensitivity. It also confirms that intrinsic plasticity is responsible for the emergence of the *input-sensitive* dynamic regime in SIP-RNs.

### Volumes of Representation

Now that the dynamic regimes of trained networks with the three combinations of synaptic and intrinsic plasticity are identified, we next move to formulating the notion of representations inside the input-sensitive dynamic regime. Developing such a notion allows linking the theory of nonautonomous dynamical systems to a theory of spatiotemporal computations. To this purpose, we coin the term *volumes of representation*, which is a concept that describes the response of a nonautonomous dynamical system in respect to its drive. The volume of representation of some input sequence within some dynamic regime is the set of network states that are *accessible* through exciting the network with the corresponding input sequence, starting from all network states in this dynamic regime as initial conditions (Definition 10). The *order* of a volume is defined by the length of the input sequence it represents. We also introduce the *volumes' inclusion property* which hierarchically links the system's response to spatiotemporal input sequences to their sub-sequences.

To visualize a network's volumes of representation, we sample the network's response. We do this because the size of the state space and the input-sensitive dynamic regime is too large, making a complete coverage impossible. Also, since volumes of representation can have complicated shapes in both the full and reduced state space, we approximate these volumes with ellipsoids.

Figure 5D provides such an approximation to the volumes of representation of order-1. The sample is a single sequence of 10000 Markov-85 inputs to a SIP-RN. Each volume is replaced by an ellipsoid. The center of this ellipsoid is the coordinates' average of the visited network states in the principal components space. Each of its semi-axes has a length that is the standard deviation from the mean of the corresponding coordinate. Also, according to the *volumes' inclusion property*, stated formally in the Methods section, a volume of representation of order-1 of some input 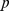 includes all volumes of order-2 for sequences whose most recent input is 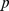. As such, Figure 5E, that depicts a similar approximation to all volumes of order-2, is also a better approximation to volumes of order-1. In Figure 5E, each order-1 volume consists of four order-2 volumes that are color-coded to match the rougher approximation in Figure 5D. In a supporting figure, we further show that this way of presentation is sufficient, compared to using percentiles of bootstrapped network states (see Figure S1).

The volumes of representation provide a geometric view of spatiotemporal computations as the ability of the recurrent neural network to *represent* in its activity, in other words to *encode*, useful functions of the network's input sequences, and for these representations to be distinguishable and reliable. In the case of the tasks RAND x 4 and Markov-85, the functions that the network activity represents are the identity, delayed or forecast. As shown in Figure 5D–E, the volumes of representation of SIP-RNs under Markov-85 input exhibit higher *separability*, which explains both their high classification performance and high mutual information. One also notices that the volumes of representation of order-2 that belong to the most probable transitions in the Markov-85 input, e.g., 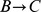, are also the most distant from one another (Figure 5E). This results in the most probable transitions to be more easily distinguishable by optimal linear classifiers.

In order to isolate the roles of synaptic and intrinsic plasticity in generating useful representations, we show in Figure 5A the order-1 volumes of representation of an IP-RN in response to Markov-85 input. Compared to the SIP-RN, these volumes are highly overlapping, which explains the lower classification performance. Also, the low mutual information between the network state and the input (Figure 3) can now be explained by various network states belonging to multiple volumes of representation, at once. Also, many network states represent the same single input which is a signature of *redundancy* resulting from IP. These observations point towards STDP being the source of separability of representations in SIP-RNs, in addition to learning the structure of the input through situating the representations of the input's most probable transitions at further distances from one another.

In the case of the task Parity-3, the function that the network activity needs to represent is the sequential *exclusive or* operation over three successive binary inputs. As such, within the input-sensitive dynamic regime, two volumes of representation exists, each encodes one outcome of the nonlinear task Parity-3. According to Definition 10, these volumes are formed from an appropriate union of order-3 volumes of representation of the binary input. We provide an illustration of these two volumes of representation in Figure S2. Here also, STDP provides the separability that allows these representations to be distinguishable, while IP gives the possibility of an input-sensitive and redundant regime to emerge, and, aided by STDP, for the volumes of representation to expand.

### Attractor Landscape

The presence of dynamic regimes entails the existence of *attractors*, i.e. limit sets of the dynamics, that apply a pulling force on the dynamical system's activity and dictate its course of flow. In an input-driven dynamical system, attractors are not easily defined as sets of states. Instead, *nonautonomous attractors* are input-dependent moving targets of the dynamics, which adds a temporal aspect to their definition (see Definition 8). As follows, for our nonautonomous dynamical systems theory of spatiotemporal computations to be complete, we link the geometry of the computational entities, i.e. the volumes of representation, to the geometry of the nonautonomous attractors. This allows us to connect the features of the volumes of representation emerging from plasticity, namely, separability and redundancy, to the effects of plasticity on the nonautonomous attractor. To that end, starting from the volumes of representations, we define the *perturbation set* (Definition 10) as a moving source of the neural activity towards its moving target, the nonautonomous attractor. Since the perturbation set changes with time, it is called a *nonautonomous set* (Definition 7). This also applies to nonautonomous attractors. The set of states constituting a nonautonomous set at a fixed time 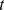 is called the set's *t-fiber*. We later show how the t-fibers of these nonautonomous sets relate to each other.

In the input-insensitive dynamic regime, the dynamical system behaves as an autonomous dynamical system, and so does its attractor, which is the period-4 attractor in Figure 5B–C. In addition, the existence of a *nonautonomous basin of attraction* (Definition 9), that constitutes the input-sensitive dynamic regime in SIP-RNs, necessitates the existence of a *nonautonomous attractor*.

It is not possible to fully identify the nonautonomous attractor by looking into the nonautonomous dynamics. This is because the attractor is not fixed in space and because the dynamics almost never converges to it. However, we prove in Theorem 11.1 that in an input-driven discrete-time dynamical system, and within a basin of attraction, the nonautonomous attractor is a subset of the basin's perturbation set, and that the t-fibers of a nonautonomous attractor are subsets of the t-fibers of the perturbation set. Given this result, the location of the nonautonomous attractor within the state space of the network can be approximated by the perturbation set. The perturbation set summarizes how the network activity passes from one volume of representation to another, at every time step, according to the input's transition statistics. We replace the time dimension in Figure 5D by arrows that correspond to the transitions in Markov-85 input. The volume of representation visited at time 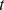 is the volume corresponding to the input at that time, and it forms the t-fiber of the perturbation set.

Instead of defining the asymptotic dynamics of the model neural network within the input-sensitive basin of attraction by a single nonautonomous attractor with different t-fibers, we can define it by multiple autonomous attractors, each belonging to a particular input. According to Theorem 11.2, within the input-sensitive basin of attraction, there exists for each input 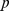, an autonomous attractor (Definition 4) of the autonomous semi-dynamical system defined by 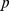. The theorem also shows that this attractor is a subset of the volume of representation of 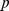. Theorem 11.3 further shows that the basin of attraction of the autonomous attractor is also the input-sensitive basin. Accordingly, the network dynamics undergoes a *bifurcation* at each time step the input changes its identity. A bifurcation is a change in the topological properties of invariant sets, such as attractors. We observe bifurcations in the input-sensitive regime of kWTA networks. The topological property undergoing the change is the loss of stability of the periodic attractor associated with an input 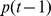, and the appearance of an attractor with a different period and location that is associated with the input 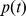.

Figure 5F shows the autonomous periodic attractors associated with each Markov-85 input within the input-sensitive basin of attraction of a SIP-RN. Each of these attractors is also a t-fiber of the nonautonomous input-sensitive attractor. While these autonomous attractors are depicted in one state space, overlaying them in a single plot serves only in illustrating the geometric relations between them. In reality, these attractors do not *coexist*. Each autonomous attractor appears in the phase space of the network when its associated input drives the network, and the attractor from the previous time step disappears.

The geometry of the nonautonomous attractor within an input-sensitive dynamic regime is very important regarding spatiotemporal computations. In fact, computations are completely defined according to the relative positions of the nonautonomous attractor's t-fibers to one another, and to the volumes of representation. An attractor consists of limit points of a basin of attraction. Thus, it exerts a pulling force on the network states that define the volumes of representation. So, if the t-fibers of a nonautonomous attractor are close to one another in the state space of the network, different volumes will be overlapping and computations will be difficult to carry through. Such is the case in IP-RNs. On the other hand, distant t-fibers of the nonautonomous attractor result in separate volumes of representation and better spatiotemporal computations, which is the case in SIP-RNs (Figure 5D–F). Also, the number of states comprising the t-fibers of the nonautonomous attractor effects the redundancy of representations. As intrinsic plasticity increases the number of states of these t-fibers, the perturbation set becomes more redundant. Given the above, while the perturbation set contains the nonautonomous attractor, it is the attractor that defines how the perturbation set, and as a consequence the volumes of representation, extends in space.

For a correct characterization of spatiotemporal computations according to the geometry of the nonautonomous attractor and function representations, we borrow the concept of *meta-transients* [44]. A transient activity of an autonomous (semi-)dynamical system is the trajectory its dynamics follows as it approaches a fixed attractor. Alternatively, an attractor of an input-driven dynamical system changes constantly. This leads the trajectory pursued by the dynamics to switch its course, so as to keep track of its moving target. Such an input-dependent trajectory is termed a meta-transient. When the input changes, the meta-transient passes from one volume of representation to another, i.e. the dynamics bifurcates and the meta-transient approaches the vertexes of the current attractor, while being repelled from the others that are now unstable. It is in this geometric relation to the different attractors (or t-fibers) that computation resides. In fact, as a proof of principle, the autonomous attractors of SIP-RNs were allocated. This was done by clamping each input for a sufficient time until the dynamics converges to that input's periodic attractor. Then, optimal linear classifiers were fitted to perform the three computational tasks. As training data, the Hamming distances between the meta-transient and the vertexes of these periodic attractors were used. Figure S3 shows the performance resulting from this computational procedure, which outperforms both SP-RNs and IP-RNs. While the performance is far from what is achieved directly from the activity of SIP-RNs, especially in the nonlinear task Parity-3, it is important to note that distance is a very rough compression of the geometric relations between the meta-transient and the autonomous attractors. For instance, distance does not allow the distinction between network states that are symmetrical in relation to the autonomous attractors.

### Emergence of Computation

We now outline how the interaction of homeostatic and synaptic plasticity gives rise to spatiotemporal computations through developing useful representations. To this end, we combine the analysis of dynamic regimes, volumes of representation, and autonomous and nonautonomous attractors (Figure 5) with the informational-theoretic intuitions regarding the evolution of the neural code (Figure 3).

At the beginning of the plasticity phase, STDP has the upper hand and it generates a minimal code of the input. This is evident from the 2 bits network state entropy (Figure 3A) and the close to zero mutual information with input (Figure 3B) at the beginning of the plasticity phase of SIP-RNs. The minimal code captures, through an input-insensitive periodic attractor, the most probable transitions in the input (Figure 5B). Another feature of the input-insensitive periodic attractor is the high separability of its vertexes in the state space of the SIP-RN.

At the same, IP time succeeds in reducing the excitability thresholds of some neurons, such that more network states become accessible at the vicinity of the vertexes of the input-insensitive attractor: entropy increases alongside the potential for redundancy. STDP concurrently assigns these network states to the inputs that induce them: mutual information and redundancy increase. This incremental process manifests dynamically in the appearance of the input-sensitive basin of attraction, and the associated appearance and expansion of volumes of representation (Figure 5D–E). Due to the highly separate vertexes of the input-insensitive attractor and the neighborhood relations of the volumes with these vertexes, the volumes of representation are highly separate. This shows that the input-insensitive dynamics is a necessary prerequisite for the emergence of spatiotemporal computations, as it sets the stage for the appearance of separate representations that also carry the structure of the input.

The emerging dynamics can also be viewed through formulating the SIP-RN during the plasticity phase, as an input-driven dynamical system parametrized by the weights and the excitability thresholds. Through varying the parameters of the system with STDP and IP, the dynamics at some point in the parameters space bifurcates from one stable dynamics, the input-insensitive dynamics, to two stable dynamics with the appearance of the input-sensitive attractor in whose basin computations are realizable. This also applies to each member of the family of semi-dynamical systems with the appearance of new dynamics and the associated new periodic attractor (Figure 5F).

### Noise-Robustness

Equipped with different vantage points to describe the information processing properties of plastic recurrent neural networks, we now turn to ask a central question: what does an information processing system like the brain require in order to be noise-robust? We state the following hypothesis. Noise-robustness is an effect of the interplay between 1) a *redundant code* that provides multiple possible encodings of an input, and 2) *separability* of representations which allows for a *margin of noise* without obscuring the identity of the input.

The analysis of the neural code (Figure 3) shows how IP increases the potential for redundancy by increasing the neuronal bandwidth. STDP could exploit this potential redundancy by assigning multiple neurons to the same input. Viewing the network dynamics in the principal components space, on the other hand, made clear that STDP ensures separability in the volumes of representation (Figure 5D–E). This also suggests that the recurrent network should be more robust to noise, the more recent the decoded input is, as the margin of noise becomes smaller for older inputs. The expansion of volumes of representation in IP-RNs also points towards a higher potential redundancy.

**Figure 6:**
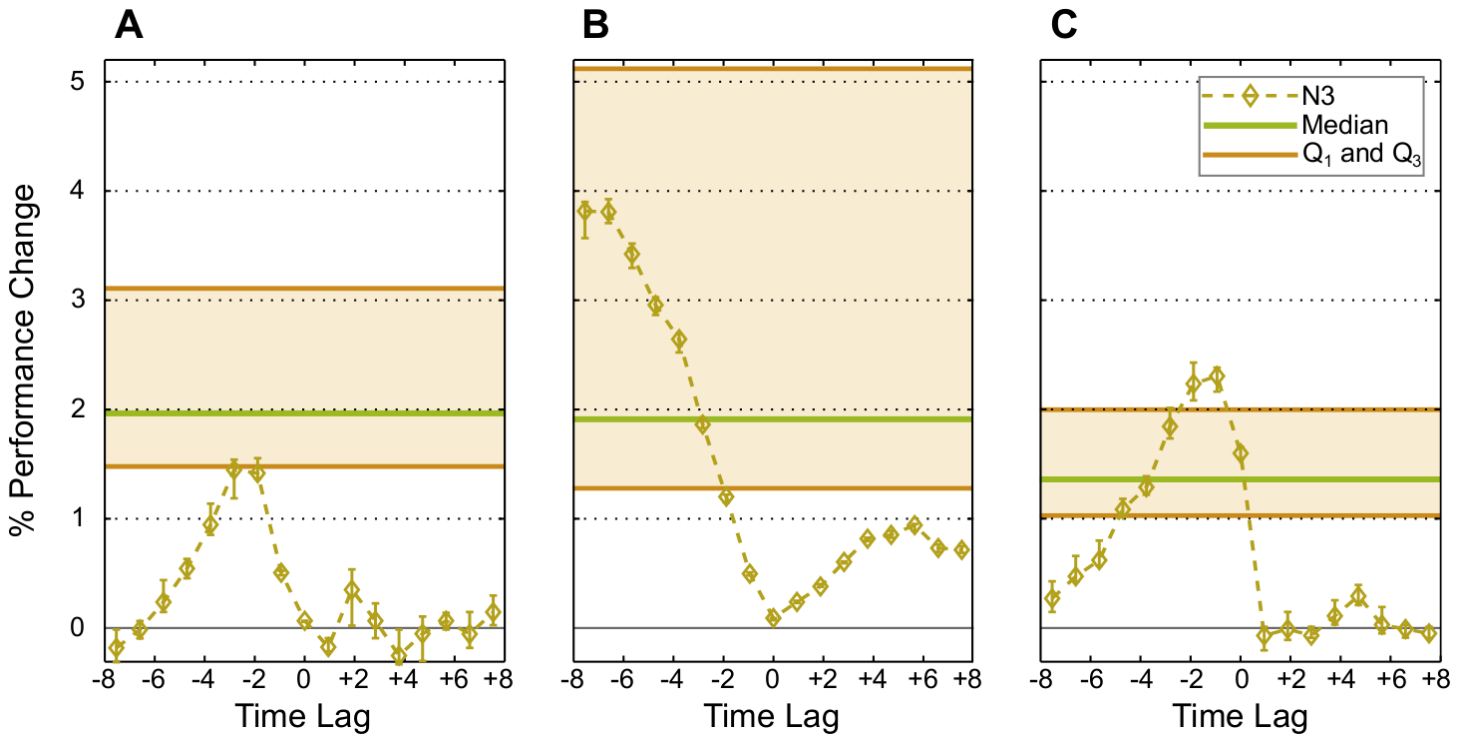
Noise-robustness is achieved through the interaction of synaptic and intrinsic plasticity. Bootstrapped median relative change from the noiseless performance of 100 networks trained with both STDP and IP on (A) the memory task RAND x 4, (B) the prediction task Markov-85, and (C) the nonlinear task Parity-3. High perturbation of 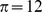 is applied at the end of the plasticity phase. Error bars correspond to the 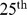 and the 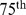 percentiles. Noise level 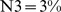 is the probability of a bit flip in the network state, that is, the probability of one of the 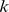 spiking neurons at time step 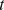 to become silent, while a silent neuron fires instead. The shaded area indicates the ratio of noisy spikes which is measured in comparison to the noiseless SIP-RNs. The green line indicates the median and the orange lines the 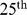 and the 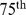 percentiles of the noisy spikes ratio.

We test the hypothesis and the role of STDP and IP interaction in noise-robustness by injecting nondeterministic noise into the recurrent network. Following the plasticity phase, we deploy a certain rate of random bit flips on the network state that reserves the kWTA dynamics, i.e. if some neuron is silenced due to noise, another neuron is selected at random and it fires instead. Different networks with different input statistics will amplify the same amount of noise to a varying extent. The shaded area in Figure 6 marks the ratio-of-noisy-spikes range within the network states of 100 recurrent networks. For all tasks and networks, we measured performance of optimal linear classifiers on both the noise-free and noisy network states, and computed the relative change in performance.

We compare the change in performance for each time-lag with the ratio of noisy spikes. To understand how this comparison aids in characterizing noise-robustness, we rely on an example. If 10% of a network's spiking activity has been replaced by noise, spikes being the carriers of information, 10% of the information in the network would be lost. However, if the activity of other neurons within the network is a replica of half the lost spikes, only 5% of the information would be lost, and the performance of the linear classifiers would decrease just as much. Having the change of performance below noise level is evidence of noise-robustness due to redundancy and intrinsic plasticity.

Information carried by the network cannot deteriorate beyond the amount of noise; the ability to perform computations, on the other hand, is another story, since distinguishing between representations is a necessary condition for computation. Noise can lead to an overlap in the volumes of representation, which hinders the information processing capability of the recurrent neural network, since overlapping representations are indistinguishable and prone to over-fitting by decoders, linear or otherwise. However, when volumes of representation are well separated due to STDP, and redundancy is at play, performance will not exceed the amount of noise in the network: noise-robustness is still achieved.

Figure 6 shows that redundancy and separability are assuring noise-robustness in the three tasks. The effects are the strongest for the task RAND x 4. The change of performance never exceeds the range of noise for all time-lags. The change of performance on the task Markov-85 remains below the range of noise for few time-lags in the past and it remains within the bounds of the noise range for older stimuli. The networks then are still capable of tolerating noise, while the volumes of representation are becoming more overlapping. The decrease of noise-robustness for larger time-lags in the past confirms our suggestion that volumes of representation become less separate for older inputs. The analysis of order-2 volumes of representation (Figure 5E) also suggests that less probable transitions of the input are more prone to noise. This, however, was not tested. The task Parity-3 is noise-robust for 0-time-lag only and with the change in performance being within the noise range. This is understandable, since for each time-lag, order-3 volumes of representation and the associated volumes of the Parity-3 function should be separate and redundant.

These observations confirm our hypothesis that redundancy and separability are the appropriate ingredients for a noise-robust information processing system, such as our model neural network. These properties being the outcome of STDP's and IP's collaboration, suggest the pivotal role of the interaction between homeostatic and synaptic plasticity for combating noise.

### Constructive Role of Noise

Now that we have demonstrated the contributions of STDP and IP in combating noise, we turn to investigating noise's beneficial role. We have seen that perturbation at the end of the plasticity phase provides a solution to the network being trapped in an input-insensitive regime. Besides viewing perturbation as a form of one-shot strong noise, which is, biologically speaking, an unnatural phenomenon, what effect would a perpetual small amount of noise have on the dynamics of the recurrent neural network?

We again deploy a certain rate of random bit flips on the network state that reserves the kWTA dynamics. Unlike the previous section, we do not restrict noise to the training and testing phase, but apply it also during the plasticity phase. We also do not reset the network activity after the plasticity phase, i.e. the perturbation parameter 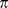 is set to 0.

**Figure 7:**
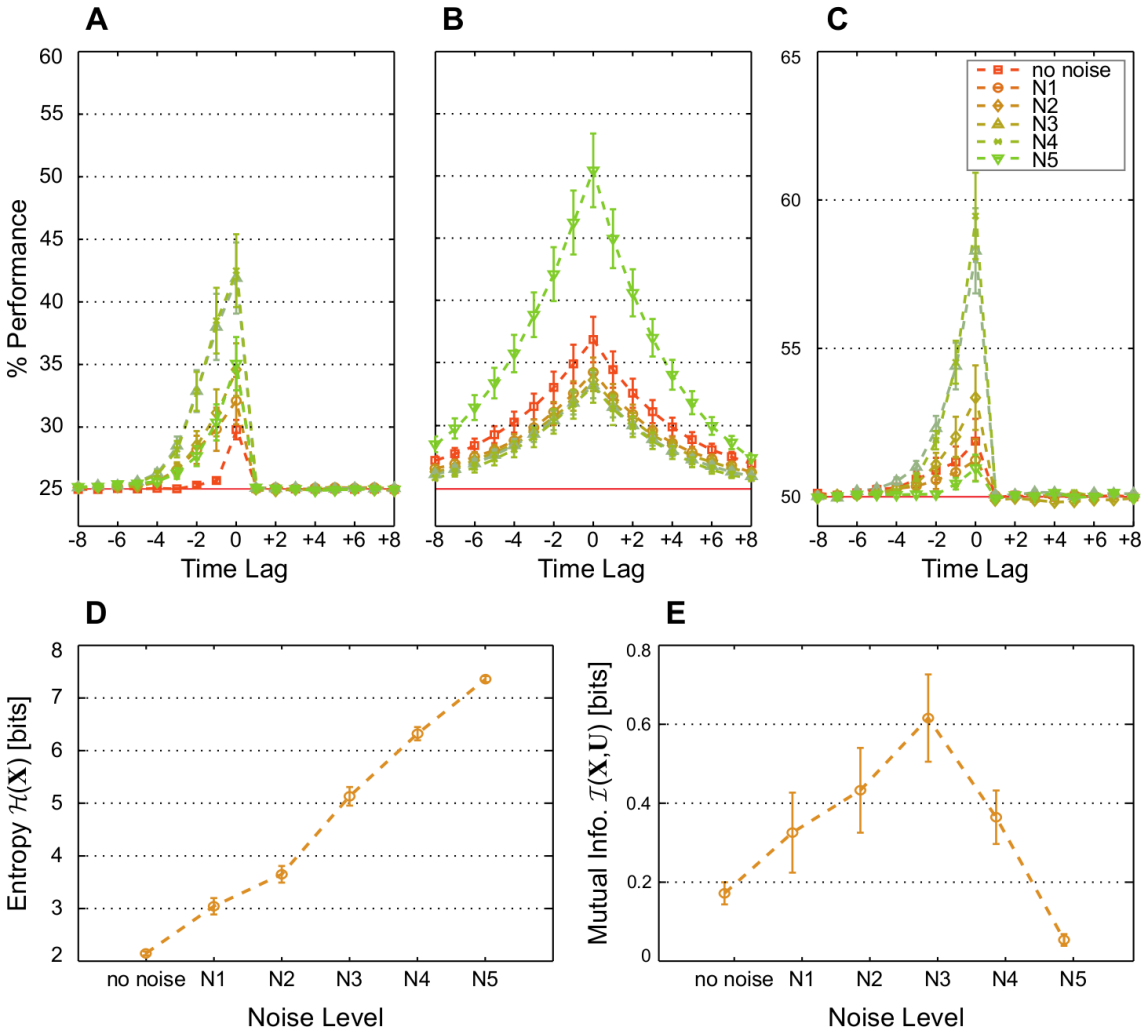
Noise at certain levels is rendered constructive when synaptic and intrinsic plasticity interact. Average classification performance of 100 networks trained with both STDP and IP on (A) the memory task RAND x 4, (B) the prediction task Markov-85, and (C) the nonlinear task Parity-3 for increasing levels of noise and no perturbation at the end of the plasticity phase (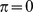). (D) Network state entropy 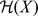 and (E) the mutual information with the three most recent RAND x 4 inputs 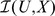 at the end of the plasticity phase for different levels of noise. Values are averaged over 50 networks and estimated from 5000 samples for each network. (A–E) Noise levels are applied during the plasticity, training, and testing phases. They indicate the probability of a bit flip in the network state, that is, the probability of one of the *k* spiking neurons at time step 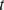 to become silent, while silent neuron to fire instead. 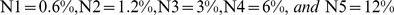. Error bars indicate standard error of the mean.

Figure 7A–C compares the performance of optimal linear classifiers on the three tasks for different levels of noise. For all tasks, some levels of noise resulted in a significantly higher average performance than the noiseless case. The task Markov-85 had the highest average performance at the largest level of noise, while the tasks RAND x 4 and Parity-3, where the input was uniformly random, had the highest performance at the third and fourth levels of noise, and the average performance dropped substantially at the fifth level of noise. In all tasks, performance was far off the levels it reached in the noiseless case (Figure 2).

**Figure 8:**
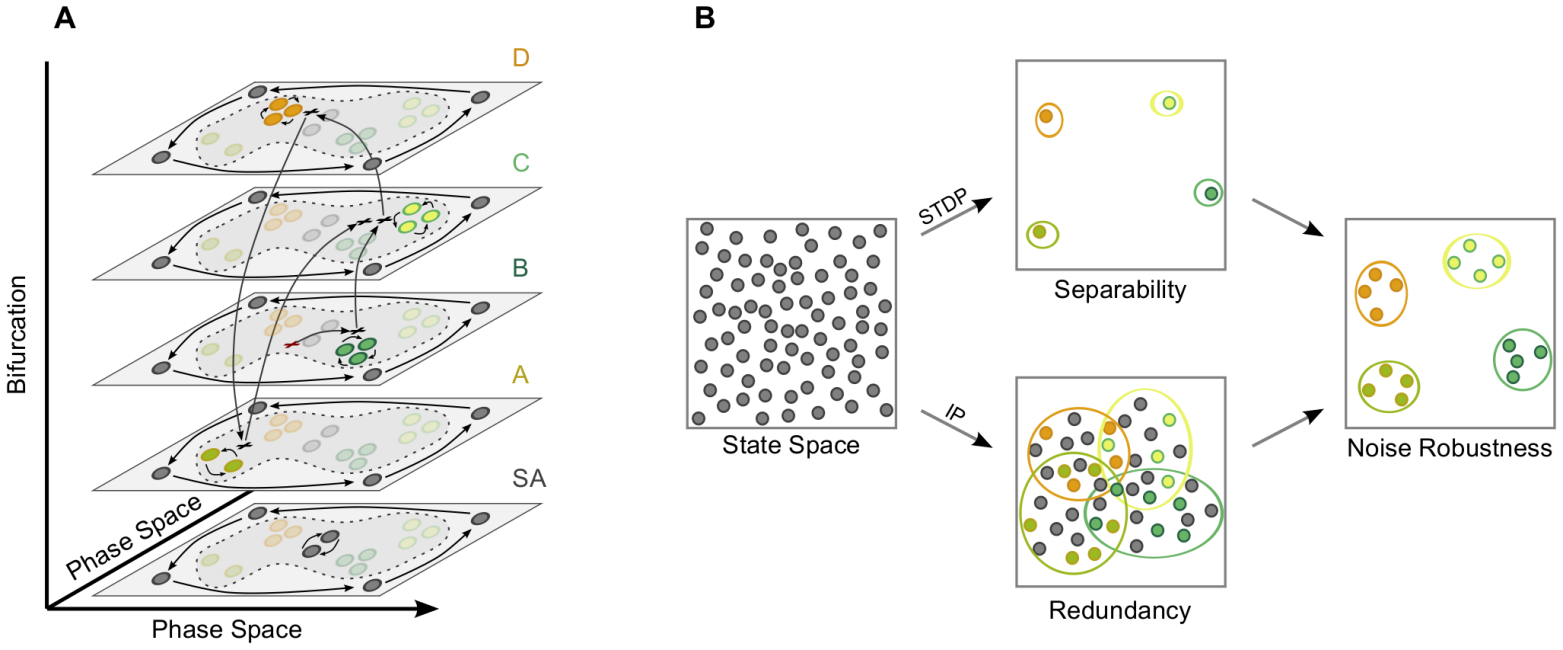
Schematics of the driven dynamics of networks endowed by synaptic and homeostatic plasticity, and the emergence of noise-robust spatiotemporal computations. (A) The dynamics of a recurrent network that is trained by homeostatic and synaptic plasticity and driven by a Markovian input. Each layer corresponds to one input. The layer illustrates a two-dimensional projection of the phase space of the autonomous (semi-)dynamical system associated with that input. A layer that corresponds to the spontaneous activity (SA) is added for completeness. Due to the interaction of synaptic and homeostatic plasticity, each of these (semi-)dynamical systems has two dynamic regimes: an input-insensitive dynamic regime that is shared by all the layers and that captures the temporal structure of the input, and an input-sensitive dynamic regime that contains a single periodic attractor. The input-sensitive attractor depends on the layer and is close to one of the vertexes of the input-insensitive attractor. The network is excited by the exemplary input sequence 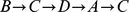. The red cross refers to the initial conditions that are chosen within the input-sensitive dynamics. Given the input sequence, the network dynamics follows the meta-transient that is illustrated by the arrows between the different layers. For instance, when the network is excited by the input *B*, the network activity approaches the *B*-attractor within the corresponding layer. When *C* follows, a bifurcation occurs, where the *B*-attractor becomes unstable and the *C*-attractor becomes stable. The meta-transient approaches the *C*-attractor from the direction of the unstable *B*-attractor. When *C* is preceded by the less common input *A*, the *C*-attractor is approached differently, such that the distance to it is bigger than in the case of the most common transition 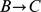. (B) Noise-robust computations are a result of the interaction between synaptic and homeostatic intrinsic plasticity. Synaptic plasticity leads to high separability and intrinsic plasticity to redundancy. These effects lead to a neural code that allows a higher margin of noise and alternative representations of computations, thus facilitating noise-robustness.

Information-theoretical quantities are again measured on networks with RAND x 4 input. As expected, the network state entropy increases monotonically with noise (Figure 7D). Mutual information, on the other hand, starts dropping for noise larger than the third level (Figure 7E). This is also expected from the change of performance (Figure 7A). Noise then appears to provide, in some of the SIP-RNs, the necessary means to escape the input-insensitive dynamics. At some levels, however, the network activity becomes dominated by noise beyond the compensatory effects of redundancy and separability achieved through plasticity. In addition, more unstructured noise during the plasticity phase delays the creation and expansion of useful volumes of representation, thereby hindering computations further.

## Discussion

We demonstrated how the interaction of synaptic learning and homeostatic regulation boosts memory capacity of recurrent neural networks, allows them to discover regularities in the input stream, and enhances nonlinear computations. We provided a geometric interpretation of the emergence of these spatiotemporal computations through analyzing the driven dynamic response of the recurrent neural network. We view computations as a geometric relationship between *representations* of functions over stimuli, representations that consist of network states, and the asymptotic dynamics of the network, i.e. attractors. Accordingly, Figure 8A shows a possible driven-dynamics viewpoint on computation, which is the following. As the stimulus changes, a bifurcation occurs where the current attractor of the network becomes unstable, while another stabilizes according to the current stimulus. That leads the network dynamics to change its course towards the new stable region, or attractor, of the state space, and away from the previous attractors that are all unstable. As such, this path of the network activity, i.e. the meta-transient [44], is defined by both the stimulus sequence and the locations of the network's attractors. Together, they lead the meta-transient to pass through particular representations which encode computations. An equivalent alternative to the *chain of bifurcations* between autonomous attractors is that of a single *nonautonomous attractor* that behaves as a stimulus-dependent moving target of the dynamics.

We showed that a successful implementation of these spatiotemporal computations requires the interaction of synaptic and homeostatic intrinsic plasticity which generates *useful representations* in the dynamics of excitable cortical networks. Figure 8 schematically illustrates the stimulus-driven dynamical viewpoint of spatiotemporal computations and the effects of plasticity. Synaptic plasticity produces stimulus-insensitive dynamics that captures the temporal structure of the input. Intrinsic plasticity increases the neuronal bandwidth by increasing sensitivity to stimuli, which reduces the dominance of the stimulus-insensitive dynamics. This, in combination with synaptic plasticity, generates stimulus-sensitive attractors and *redundant* representations around them. These stimulus-sensitive components are pulled apart by the stimulus-insensitive dynamics, so that the structure of the input is preserved, and the *separability* of representations is higher and computations are realizable.

We pointed out throughout the text that computation is an *emergent* property of the recurrent network, and that it cannot be fully understood from the individual contribution of the parts, be it neurons or plasticity mechanisms. It might appear contradictory to that statement that the analysis was often concerned with the isolated role of each single plasticity mechanism. However, the quantitative assessments of computations point back to the emergent and collective aspect of computation. Namely, measured on SIP-RNs, neither performance of linear classifiers nor mutual information with input can be accounted for by a linear relationship between the respective quantities measured on SP-RNs and IP-RNs. In fact, the performance of networks where the recurrent weights and firing thresholds are adapted separately, and then combined following the plasticity phase, is far less than the performance of SIP-RNs, where intrinsic and synaptic plasticity are mutually active (see Figure S4). This further consolidates the claim that computations in SIP-RNs *emerge* from the *interaction* of STDP and IP, and *not* from their isolated contributions. It also points back to the formation of separate and redundant representations from the continuous interplay of these two mechanisms.

We also illustrated the combined role of synaptic and homeostatic intrinsic plasticity in creating noise-robust encoding through the generation of a redundant neural code. Many studies have investigated the redundant nature of neural information transmission in many cortical regions, and have justified this expensive allocation of neural recourses by redundancy serving as an error-correction strategy that provides neural assemblies with the capacity to average out noise [10], [47]–[50]. Tka
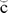
ik and colleagues have shown that in the presence of noise, a maximum entropy model of the retina increases redundancy for higher noise levels. A side effect of their model is that stimulus representations become highly separate, which increases the tolerance margin of noise and enhances information transmission [51]. Our model was able, through *local* plasticity mechanisms, to capture both of these properties, achieved in [51] through optimality principles, and to lead to a noise-robust population code (Figure 8B). Namely, synaptic plasticity enhances the separability of representations through the pulling force of the input-insensitive attractor, while intrinsic plasticity perturbs the network states and increases redundancy when interacting with synaptic plasticity, which allows for alternative representations of similar input sequences. Another point of similarity with the model of Tka
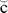
ik and colleagues [51] and with empirical findings [52], [53] is the remnant fingerprint of the most common stimulus in the network's spontaneous activity, which manifests in our model neural network in the stimulus-insensitive dynamics (Figure 5B–C).

In addition to combating noise, our model explores a potential benefit from its presence. We pointed out the necessity of the stimulus-insensitive dynamics for the emergence of computation in the model neural network. The stimulus-insensitive attractor provides the baseline dynamics for the appearance of highly separate representations, and thus, the excitable dynamics necessary for computations. Getting from the input-insensitive regime to the excitable one depended, however, on the *ad hoc* reinitialization of the network activity at the end of the plasticity phase. Noise provides an alternative. During the plasticity phase, noise shallows the boundaries between the two basins of attraction, which reduces the dominance of the stimulus-insensitive attractor. After the plasticity phase, noise supplies the small perturbations needed to get the network activity to the sensitive dynamics where computations are possible. This solution, in comparison to reinitializing the network activity, is more inferior, specifically because noise also delays the learning of representations. We postulate that another homeostatic plasticity mechanism, *synaptic scaling*, might contribute to the shallowing of the attractor boundary by constraining the strength of synapse bundles between neural subpopulations (e.g., between 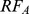 and 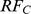). For instance, synaptic scaling was necessary for implementing spatiotemporal computations in ***s*** *elf-* ***o*** *rganizing* ***r*** *ecurrent* ***n*** *etworks* (SORN) [28], but no analysis of the dynamics of these networks was done. Testing this hypothesis is, however, beyond the scope of this work.

It is also tempting to connect the topology of the attractor landscape of SIP-RNs to neuropathology and to a model by Pfister and Tass [54]. They suggest that two stable regimes of recurrent network activity, a synchronous pathological regime and an asynchronous healthy regime, coexist, and that their coexistence is a necessary condition for the functioning of a model of deep brain stimulation. In their model, the stimulation of the recurrent network destabilizes the synchronous dynamics through inducing STDP. The destabilization drives the network activity towards the healthy asynchronous basin of attraction. By eliminating the stimulation, the energy hill between the two dynamic regimes rises again and the network remains in the healthy dynamics. Our study has shown how these two coexisting dynamic regimes and their associated forms of activity might come into being through neuronal plasticity. We also suggested noise as a possible mechanism for avoiding the unhealthy dynamics. Further analysis is necessary to investigate how the interaction between noise and different plasticity mechanisms might contribute to our understanding of neurological disorders.

Our analysis of spatiotemporal computations was restricted to Markovian dependencies in the temporal structure of the stimulus or to no dependencies at all. This is often not the case in natural stimuli faced by animals and humans, where the Markov property does not always hold. Lazar et al. have shown that SIP-RNs are capable, to a certain degree, of performing predictions on second-order Markov chains [27]. However, optimal encoding of non-Markovian stimuli and performing computations over them require forms of spike-timing-dependent plasticity that are less myopic to the temporal dependencies than what we considered in this work (Figure 1B). For instance, Brea and colleagues have shown that storing and reproducing a non-Markovian sequence in a recurrent neural network require a nonlocal form of STDP with more complex temporal dependencies between pre- and post-synaptic spikes [55]. While their model was not concerned with carrying through spatiotemporal computations of the kind we presented here, it successfully reproduced the stored non-Markovian input in the spontaneous activity of the neural network. This refers to a point of similarity to the simpler case we presented here, where Markovian input was stored and recalled in the spontaneous activity of the input-insensitive dynamics. In any case, while spatiotemporal computations over non-Markovian stimuli and the necessarily more complex plasticity mechanisms that lead to their emergence, are not considered here, we view the concepts and methodology developed above as a general framework for future studies.

In this article, we provided a first analysis of the combined role of synaptic and intrinsic plasticity on the emergent dynamics of recurrent neural networks subject to input. We redefined computations in relation to these emergent dynamics and related that to properties of the neural code. We also considered how the neural dynamics interact with noise, both as a nuisance to combat, and as a driving force towards healthy neural activity. The model we used is simplified, however, both in network architecture and plasticity mechanisms. While this simplification is necessary for mathematical convenience, biology never cares for formal abstractions, for the brain is a complex information processing system that is rich with a variety of neuronal morphologies and functions. The plastic changes the brain undergoes are neither confined to the two mechanisms we dealt with here, nor are they uniform across different regions. On the other hand, mathematical formalization of computation and adaptability allows the identification of unifying principles in computational biology, in general, and neural computations, in particular. We intended the current article as a step in that direction.

## Methods

The setup on which we assessed spatiotemporal computations in recurrent neural networks is partially inspired by the theory of ***r*** *eservoir* ***c*** *omputing* (RC) [3], [29], [30]. However, as shown in the Results section, our analysis is independent of the RC paradigm, as it is concerned with the effects of plasticity on the recurrent network, and optimal linear classifiers are only used as one possible probe to quantify these effects. We present in this section the *recurrent network* (RN) architecture and the plasticity mechanisms active in shaping the neural response. We follow by introducing the computational tasks and justifying their selection. We then specify the simulation conditions and the training of optimal linear classifiers, followed by demonstrating how information-theoretical quantities are estimated. We finally lay down the mathematical formalization of the autonomous, input-driven, and input-insensitive dynamics of the recurrent network: We adapt Definitions 2, 4, 6–8 from [31] to the special case of discrete-time dynamics [32], which is the case that concerns the current article. We contribute the new concepts of volumes of represen\r notation and purposes.

### Network Architecture

In this paper, the model recurrent network is of the *k-Winner-Take-All* (kWTA) type [27] that consists of 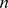 memoryless binary neurons from which only 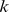 neurons are active. The discrete-time dynamics of the recurrent network at each time step 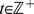 is given by
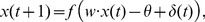
(1)where 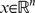 is the network state. The nonlinear function 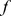 sets the 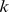 units with the highest activities to 1 (spiking), and the rest to 0 (silent). As such, the population firing rate is held constant at 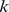, and there is no need to introduce inhibitory neurons to balance excitation and inhibition. Recurrent synaptic efficacy is defined by the weight matrix 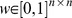 with 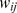 being the efficacy of the synapse connecting neuron 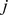 to neuron 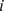. Self-coupling is avoided by setting diagonal elements 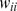 to 0. 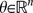 defines neuronal firing thresholds that modulate the neurons' resistance to firing, and hence, their excitability. 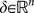 is the external drive whose dynamics depends on the task performed.

More formally, the set of possible network states is a metric space:

**Definition 1.** Given the set 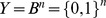 of all binary vectors of size 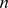, we define the *Hamming metric* by the function:
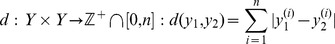



According to this metric, the distance between two vectors of 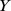 is the number of bits at which these two vectors differ. The Hamming metric is a proper metric on strings of fixed length which is the case for 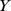. The pair 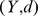 then forms a *metric space*. It is also equivalent to the 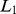 norm on the set *Y*, which allows us to define the *Hamming length* of a vector 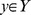 as the Hamming distance between 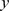 and the 0-vector, i.e. 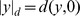.

Given the kWTA dynamics (see Equation 1), the network activity is restricted to the set:
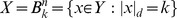
(2)


Since 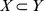, the pair 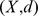 forms a metric space as well. Distances between subsets of 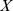 can be measured using the Hausdorff metric, which we also denote 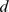.

### Plasticity Mechanisms

We are concerned with the interplay of two forms of plasticity in enhancing the computational capability of the model recurrent network.

***S*** *pike-* ***t*** *iming-* ***d*** *ependent synaptic* ***p*** *lasticity* (STDP) is a set of Hebbian/anti-Hebbian learning rules, where synaptic efficacy is modified according to the relative firing time between pre- and post-synaptic neurons [56]. We adapted a simple causal STDP learning rule by which a synapse is potentiated whenever the pre-synaptic neuron fires one time step before the post-synaptic neuron, and is depressed when a post-synaptic spike precedes a pre-synaptic spike by one time step:
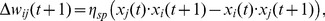
(3)where 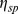 is the synaptic plasticity learning rate set to 0.001. To prevent weights from switching signs or growing uncontrollably, we enforce hard bounds such that the weights remain within the interval [0, 1].

Competition between synapses due to STDP leads to neurons with synapses that won the competition to fire consistently and those who lost the competition to be constantly silent [57]. To counteract this pathological state, the time-averaged firing rate for a neuron is modulated through homeostatic modification of its excitability threshold using ***i*** *ntrinsic* ***p*** *lasticity* (IP) [6], [7]:
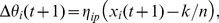
(4)where 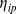 is the intrinsic plasticity learning rate set to 0.001. This rule uses subtractive normalization to pull the time-averaged firing rate of each neuron closer to the population firing rate 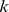.

### Computational Tasks

Neural circuits in different brain regions adapt to best serve the region's functional purpose. To that end, we constructed three tasks, each of which resembles in spirit the demands of one such canonical function. We then, under the stimulation conditions of each task, compared the performance, information content, and dynamical response of networks optimized by combining both STDP and IP with networks that are optimized by STDP alone or IP alone.

In all tasks, the network is subject to perturbation by a set of inputs 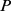. The receptive fields of non-overlapping subsets of neurons 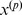 are tuned exclusively to each input 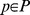. As such, each input 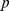 has its corresponding receptive field 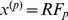 in the recurrent neural network. When an input 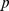 drives the network, all neurons 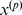 receive a positive drive 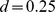, while the rest 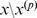 receive none. Readouts are trained on the current network state 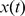 to compute a function over input sequences 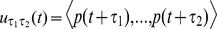, 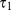 and 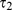 being time-lags at which target inputs are applied where positive lags corresponds to future inputs and negative lags to past ones. We restrict time-lags 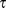 to the range 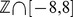.

In a first task, RAND x 4, we assessed the capacity of the network to retain memory of past stimuli within its activity. The recurrent network is driven by four randomly drawn inputs 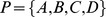. The receptive field of each input consists of 15 neurons, and one optimal linear classifier 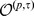 is trained for each input/time-lag pair, i.e. 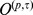 fires when 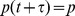 and is silent otherwise.

The second task, Markov-85, explores the ability of the recurrent network to discover temporal regularities in its input. The recurrent network receives one of four possible inputs 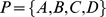 generated from a Markov chain with 85% probability for 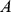 to be followed by 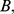 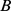 followed by 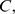 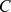 followed by 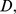 and 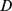 followed by 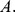 All other transitions occur with a 5% probability. Again, the receptive field of each input consists of 15 neurons, and one optimal linear classifier 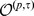 is trained for each input/time-lag pair.

With the third task, Parity-3, we exploit the nonlinear expansion provided by the recurrent neural network. Here, the network is subject to binary input 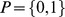, where each symbol has a receptive field of 40 neurons. The task is to identify the parity of a sequence of three successive inputs. This means that given an input sequence 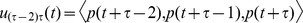, an optimal linear classifier 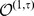 fires when 
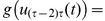
 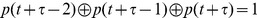, and is silent otherwise. 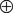 is the nonlinear *exclusive or* binary operation.

Even though every task used here is very much simplified compared to stimuli usually processed by neural systems, we would still like to link the basic properties of every task presented here to a realistic case processed by a human or an animal. The property of the memory task RAND x 4 that we want to emphasize is that a neural system must be able to process rapidly changing stimuli that are only shortly presented. That property is partly reminiscent of retinal input, which is rather stationary during moments of fixation, and rapidly changing due to saccadic eye movements. However, it needs to be noted that saccadic eye movements might be difficult to predict and may appear rather random, but are very likely structured and stimulus-dependent. This motivated the prediction task Markov-85 that models temporally structured and rapidly changing sensory input that is shortly presented. Such input could either be generated by retinal input and saccadic eye movements, or by the whisking behavior and the produced neural activity in the barrel cortex of a mouse. In addition, nonlinearities are prevailing in natural stimuli, and to highlight the necessity of processing these stimuli, we used the nonlinear task Parity-3. Such computational demands can be easily motivated by occlusion in vision, where pixel intensities do not sum up linearly at points where one object occludes another in the visual field. Again, we stress that none of these tasks is a good model of real processing in neural systems in nature. However, each is sharing individual aspects that are motivated by real life examples.

### Simulation Conditions

In order to isolate the role of STDP and IP in shaping the computational and information processing properties of the recurrent network, we compared networks trained by both STDP and IP, with networks that are trained by STDP alone or IP alone.

Throughout all experiments, we trained networks of 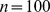 neurons on either the STDP+IP condition, the STDP condition, or the IP condition for a *plasticity phase* of 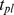 time steps. For convenience, we call a *recurrent network* trained with both *synaptic* and *intrinsic* plasticity SIP-RN. In contrast, we name a recurrent network that learned with a single plasticity mechanism either SP-RN or IP-RN. 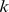 is set to 12, the initial weights are chosen uniformly on the interval [0, 0.1] with 10% connectivity probability, and thresholds are drown from a Gaussian distribution with 0 mean and 0.1 standard deviation. Under the IP condition, to assure that weights' distribution is not different from conditions where STDP modifies the synaptic efficacies 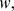 a *pre-plasticity phase* of similar length to the plasticity phase precedes the latter, where both STDP and IP are active. Afterwards, the weights structure is destroyed by random shuffling and the plasticity phase starts where IP is turned on.

In all experiments where the performance of optimal linear classifiers is estimated, the plasticity phase was 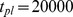 time steps long. Afterwards, weights and thresholds are held fixed, the network state is *reset* to a random initial state, and the *training phase* starts where linear classifiers are trained using linear regression on 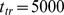 time steps, followed by a *testing phase* of performance for another 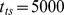 time steps.

### Post-Plasticity Perturbation

At the beginning of the training phase, the network state is reset to a random initial state. If the network dynamics is multistable, this resetting could bring it to a different regime than where the network was at the end of the plasticity phase. To test this possibility systematically, we perform the following post-plasticity perturbation.

Given some perturbation parameter 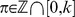. We assume the network state at the end of the plasticity phase is 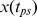. Instead of randomly choosing the initial network state for the training phase, we choose a network state 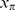 such that the condition 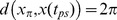 holds. To satisfy this condition, 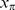 is chosen as follows. In the network state 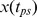, 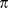 firing neurons and 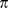 silent neurons are randomly selected. The 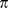 firing neurons are then silenced and the 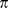 silent neurons are set to firing.

### Output Weights and Performance

According to the RC paradigm, an input signal undergoes a nonlinear feature expansion by projecting into a recurrent neural network of nonlinear units. The network recurrency also provides a sustained but damped trace of past inputs (echo state [29] or fading memory [30]) to propagate through the network. The network state is then read out by simple linear units through linear regression.

Following the plasticity phase, the network activity during the training phase
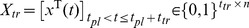
(5)provides the training data for all optimal linear classifiers, where 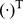 denotes matrix transpose. The target signal of output neurons for a particular time-lag 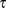 is clamped in a supervised fashion to
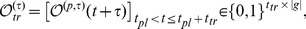
(6)where 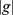 depends on the task and is the cardinality of the set of possible values which the target signal can take. 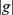 equals 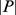 for the tasks RAND x 4 and Markov-85. Output weights 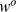 for each time-lag are then computed using linear regression through ordinary least squares
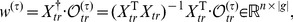
(7)where 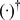 is the *Moore-Penrose pseudoinverse* of a matrix, and 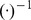 is the regular inverse of square matrices.

These optimal linear classifiers are then validated on the network activity
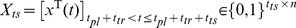
(8)during the testing phase. First, a pre-estimate of the target signal is computed for each time-lag:
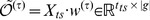
(9)


Only one output neuron fires each time step for each time-lag, and this is specified through winner-take-all on the rows of 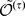. This leads to the final estimate 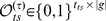. The *classification performance* for each time-lag is finally computed as the percentage of correct classifications:
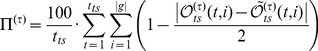
(10)


### Computing Entropy and Mutual Information

On multiple occasions, both the self-information capacity of the network state and its dependence on input was measured. Entropy measures self-information capacity which is the expected value of information carried by the network activity 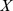 and is given by
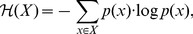
(11)where 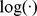 is the base-2 logarithm, so that entropy (and mutual information) are measured in bits. Mutual information measures the dependence of the network activity 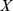 on a corresponding input sequence 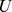 and is given by
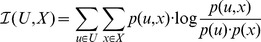
(12)


In computing entropy and mutual information, we used the algorithm and code developed in [58] that computes entropy from an adaptive k-nearest-neighbor estimate of probability density functions. This allows for reliable estimates of these quantities with far fewer samples in comparison to other algorithms. Nevertheless, due to the high number of channels we have (100 neurons), and to truncate unnecessary computation time, samples from the network activity are first transfered to the principal components space, and only components that carry 95% of the information are passed to the mutual information estimator.

We always considered inputs from the task RAND x 4 and we computed the mutual information between samples of the network state 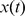 and the three most recent inputs 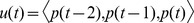. We encoded each of the four input symbols 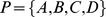 by a 3-bits code 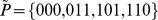 to ensure equal pairwise Hamming distances between symbols. For all cases but one, as few as 5000 samples of the network state 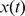 and input sequence 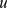 were enough to reliably estimate entropy and mutual information. The exception was computing mutual information between input and IP-RN activity, which demanded a higher number of samples (500000 time steps) and very long computation time, as covering 95% of the information required no less than 60 principal components.

### Autonomous Dynamics

For a full understanding of the emerging information processing properties of the interaction of synaptic and intrinsic plasticity, it was necessary to rely on and develop concepts from the newly emerging mathematical theory of *nonautonomous dynamical systems* [31], [32]. Throughout what follows, the correspondence of the introduced concepts to our model is clarified. First, autonomous dynamics are defined, since they form a special instance of the nonautonomous case.

**Definition 2.** Let 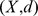 be a metric space with a metric 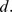 A *discrete-time semi-dynamical system* is a function 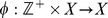 that satisfies

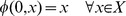
.

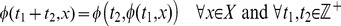
.

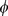
 is continuous.

Equation 1 defines the driven or nonautonomous kWTA dynamics. The autonomous alternative is given by the discrete-time difference equation
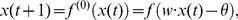
(13)where 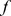 is the kWTA nonlinearity defined as above. To relate Equation 13 to Definition 2, the function 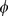 (the *solution mapping*) is chosen such that
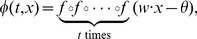
(14)where 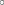 is function composition. For 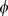 to be an autonomous semi-dynamical system, it has to satisfy the three conditions of Definition 2. The first two conditions are trivial, as they result from the definition of function composition. We turn to prove the third condition, namely, the continuity of 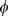. We first observe that 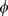 is merely the t-fold composition of the function 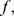 and since the composition of continuous functions is continuous, it is sufficient to prove the continuity of 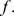


**Proposition 3.** The kWTA function 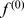 from Equation 13 defined on the metric space 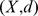 is continuous, i.e.
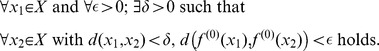



**Proof.** For all 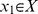 and all 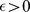, we choose 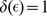. For all 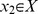, if the Hamming distance 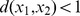, 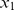 and 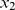 have to be equal, since the kWTA dynamics restricts the distances between any two states to the set 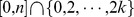. As such, since 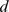 is a metric, 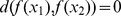, which is always smaller than 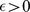. Ergo, 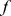 is continuous.

We note that the proof to Proposition 3 becomes trivial if we consider a result from topology which states that any function from a *discrete topological space* to another is continuous. However, the proof is interesting in that it shows that 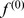 has a stronger form of continuity, that is, 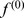 is *uniformly continuous*, since the proof shows that there exists a *packing radius* 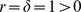 such that either 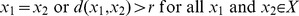.

With the proof of Proposition 3, we conclude that the kWTA autonomous dynamics in Equation 13 generates a discrete-time semi-dynamical system. A dynamical system is a semi-dynamical system with invertible dynamics, which is not the case for kWTA networks. However, for all intents and purposes, being a semi-dynamical system is sufficient for formalizing the nonautonomous dynamics of the model network.

### Autonomous Attractors

Characterizing the computational properties of the model neural network requires defining invariant sets and attractors.

**Definition 4.** Let 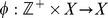 be a discrete-time semi-dynamical system generated by an autonomous difference equation 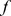 on a metric space 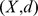. A subset 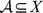 is *invariant* under 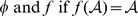, and is *positively invariant* if 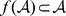. 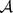 is an *attractor* of 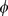 if the following conditions hold:

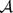
 is invariant under 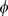 and 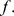

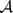
 is compact.
3. There exists a neighborhood 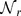 of radius 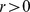 of 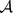 such that 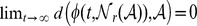

For the kWTA dynamics, the second condition is assured, since 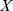 is discrete and finite, which makes all subsets compact. The third condition assures that no subset of 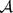 satisfies the invariance and compactness conditions. Another important concept is that of a *basin of attraction* which associates each attractor with the region of the state space that converges to that attractor:

**Definition 5.** Let 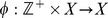 be a discrete-time semi-dynamical system generated by an autonomous difference equation 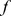 on a metric space 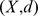. The *basin of attraction* of an attractor 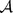 of 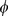 is defined by
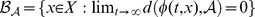



### Nonautonomous Dynamics

Unlike autonomous (semi-)dynamical systems, the elapsed time is not sufficient to find the solution for nonautonomous dynamics: both the start and end times must be specified. Accordingly, we now define a *discrete-time nonautonomous dynamical system* as a *process*. In what follows, we will make use of the set 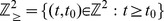.

**Definition 6.** Let 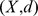 be a metric space with a metric 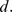 A *discrete-time process* is a function 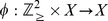 that satisfies

1. 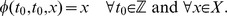

2. 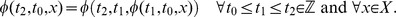

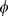
 is continuous.

We now turn to formulating the driven kWTA difference equation (see Equation 1) as a discrete-time process. We first note that for a particular task, a set of possible inputs 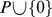 is defined. For completeness, this set covers the autonomous case by including the 0-vector. For each member of this set, we define a separate map 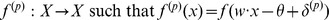. The set of maps 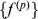 with cardinality 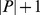 defines a family of discrete-time autonomous semi-dynamical systems. These maps are chosen either randomly for the tasks 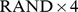 and Parity-3, or in a more structured fashion for the task Markov-85. In either case, the kWTA discrete-time nonautonomous dynamics in Equation 1 can be rewritten in the form
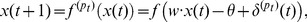
(15)which generates a solution mapping
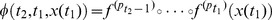
(16)


The solution mapping 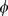 satisfies the three properties of a process. The first two properties are a product of the definition of function composition, and the continuity condition is proven exactly as in Proposition 3. Given the above, the family of discrete-time autonomous difference equations 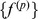 on the metric space 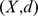 generates a process 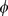, and thus, it defines a particular kind of nonautonomous dynamical systems termed an *input-driven dynamical system*.

It is important to point out that an input-driven dynamical system is not defined for a particular input sequence, but for all input sequences drawn from its input set. This becomes more explicit if one considers the alternative *skew product* definition of a nonautonomous dynamical system, where the input is treated as a driving autonomous dynamical system [31], [32]. We compare the two definitions of nonautonomous dynamical systems in Text S3. We now cover a few important concepts that will aid in defining the dynamic behavior of the model neural network.

### Nonautonomous Attractors

Attractors in nonautonomous dynamical systems are defined on *nonautonomous sets*, relating strongly to the concepts of *invariance* and *entire solutions*.

**Definition 7.** Let 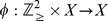 be a discrete-time input-driven dynamical system generated by the family of autonomous difference equations 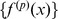 on a metric space 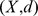. A subset 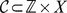 is called a *nonautonomous set*, and for all 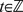, the set
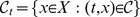
is called the *t-fiber* of 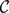. 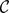 is said to be *invariant* under 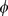 if 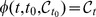 for all 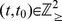. An *entire solution* of 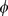 is an invariant set under 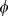 whose t-fibers are the singleton sets 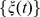 that are the images of the function 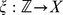 such that
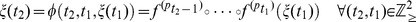



An important property of invariant nonautonomous sets is that they consist exclusively of entire solutions (for a proof, see Lemma 2.15 in [31]). Nonautonomous attractors are nonautonomous sets. As such, they consist of entire solutions as well. There are several types of attractors of nonautonomous dynamical systems. Only of interest to our model neural network are forward attractors, so we drop the qualifier ‘forward’ and substitute it with ‘nonautonomous’.

**Definition 8.** Let 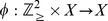 be a discrete-time input-driven dynamical system generated by the family of autonomous difference equations 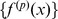 on a metric space 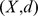. A nonautonomous set 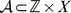 is a *nonautonomous attractor* of 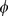 if the following conditions hold:

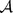
 is invariant under 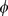.

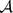
 is compact.
3. There exists a neighborhood 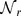 of radius 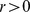 such that 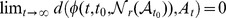 for all 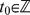

As in the autonomous dynamics of kWTA networks, all subsets of 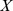 are compact. The third condition assures that no subset of 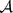 satisfies the invariance and compactness conditions. One may generalize the concept of a basin of attraction in an autonomous dynamical system to the nonautonomous case. This concept associates each nonautonomous attractor with the region of the state space that converges to that attractor:

**Definition 9.** Let 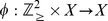 be a discrete-time input-driven dynamical system generated by the family of autonomous difference equations 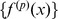 on a metric space 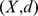. The *nonautonomous basin of attraction* of a nonautonomous attractor 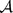 of 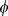 is defined by
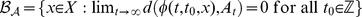



### Volumes of Representation

Spatiotemporal computations requires encoding different input sequences in the states of the neural network. The set of network states accessible from some initial conditions within a basin of attraction through perturbing the network with a particular input sequence 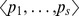 defines this sequence's *volume of representation*.

**Definition 10.** Let 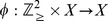 be a discrete-time input-driven dynamical system generated by the family of autonomous difference equations 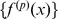 on a metric space 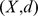. Given an input sequence 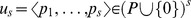 and a basin of attraction 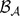, a subset
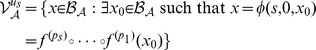
is called the *volume of representation* of the input sequence 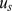 within the basin 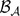. The sequence length 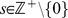 defines the *order* of this volume. The nonautonomous set 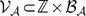 whose t-fibers are order-1 volumes of representation 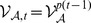 is called the *perturbation set* within 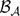. Also, given a function 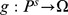 on input sequences such that 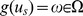, the set
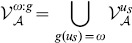
is the volume of representation of 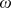 given *g*.

It is straightforward to show that, within a basin of attraction, the volume of representation of some sequence 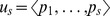 is a superset of the volume of a sequence 
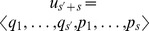
 for all 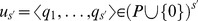, and that the volume of 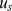 is equivalent to the union of the volumes of 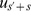 for all 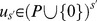. We term this property the *volumes' inclusion property*.

The concept of ‘volumes of representation’ allows us to state the following theorem on the nature of attractors in discrete-time input-driven dynamical systems:

**Theorem 11.** Let 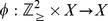 be a discrete-time input-driven dynamical system generated by the family of autonomous difference equations 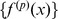 on a metric space 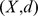, and let 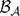 be a *compact* nonautonomous basin of attraction. The following holds:

1. The perturbation set 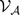 is a superset of 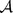.
2. Within 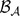, and for all 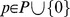, there exists one attractor 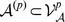 of the discrete-time autonomous semi-dynamical system generated by 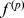.

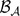
 is the basin of attraction of 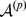 for all 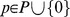.

**Proof.**

1. Since every attractor, whether autonomous or nonautonomous, is an invariant set, it is sufficient to prove that all invariant sets within a basin 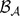 are a subset of its perturbation set 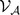. Let's consider an entire solution 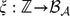. For all 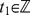, it holds that 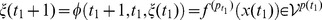. It follows by induction that 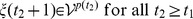. This translates to t-fibers of entire solutions being always a member of order-1 volumes of representation and that all entire solutions within a basin of attraction 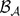 are subsets of the perturbation set 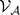. Since invariant sets consist exclusively of entire solutions, it follows that all invariant sets are subsets of the perturbation set 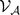, including the nonautonomous attractor 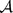.
2. Given some input 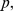 we consider the discrete-time semi-dynamical system generated by 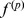 on 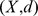 with the solution mapping
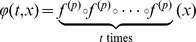
From Definition 10 of volumes of representation, the order-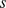 volume generated by 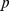 is the set 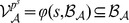. Due to the compactness of 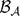 and the continuity of 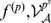 is compact for all 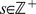. Moreover, due to the volumes' inclusion property, the family of compact volumes 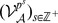 is nested with 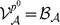. As such, and according to Theorem 1.28 in [31], there exists a nonempty set
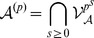
that is both *compact* and *invariant* under 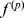 and 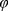. It also follows from the compactness of 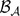 that 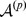 attracts 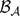, i.e. there exists 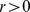 such that 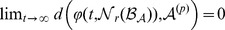, and since 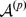 is a subset of 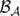, the neighborhood 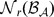 is also a neighborhood of 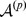. Hence, the compact and invariant set 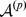 is an attractor of the discrete-time semi-dynamical system generated by 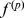 and is a subset of 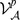.
3. Since 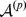 attracts 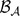, it follows that the basin of attraction of 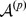 satisfies 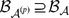. Given a point 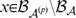, and since 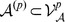, there exists 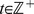 such that 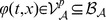, which is a contradiction, since 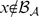. Ergo, 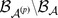 is an empty set, and 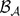 is the basin of attraction of 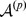.

This theorem allows us to characterize the properties and relations between autonomous and nonautonomous attractors of kWTA networks, where all subsets of 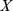 are compact due to 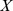
's finiteness and discreteness. Namely, it allows us, within some compact basin, to allocate the nonautonomous attractor's t-fibers as subsets of the t-fibers of the perturbation set, and it shows that the autonomous attractor of the input at time *t* is the t-fiber of the nonautonomous attractor.

### Input-Insensitive Dynamics

It is possible for a process to behave locally or globally as an autonomous (semi-)dynamical system. That is equivalent, in the case of input-driven dynamical systems, to being input-insensitive.

**Definition 12.** Let 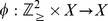 be a discrete-time input-driven dynamical system generated by the family of autonomous difference equations 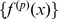 on a metric space 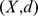. A state 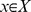 is said to be *input-insensitive* if 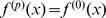 for all 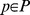. An *input-insensitive basin* is a basin of attraction that consists entirely of input-insensitive states.

This definition implies that the volumes of representation of a particular order and the t-fibers of each nonautonomous set within this basin are equivalent, including the perturbation set and the nonautonomous attractor: they reduce to autonomous sets. The *input-insensitive attractor* becomes the autonomous attractor of each discrete-time semi-dynamical system generated by a difference equation 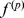.

## Supporting Information

Figure S1
**Approximating volumes of representation using percentiles.** (A) Percentile approximation of the order-1 volumes of representation of a SIP-RN. (B) Percentile approximation of the order-2 volumes of representation of a SIP-RN. Order-2 volumes are more exact approximations to the order-1 volumes according to the volumes' inclusion property. The correspondence is clarified by using similar color coding. (A,B) This approximation is done as follows. After transforming the network states to the principal components space, the coordinates of the first three principal components belonging to each volume of representation are first bootstrapped to 10000 samples, and the 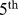 and 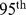 percentiles are computed. Each volume is then approximated by an ellipsoid whose semi-axes extend to these percentiles and is centered at their average. This alternative approximation is less liberal than the one that uses means and standard deviations in that it extends the ellipsoids to assure including more true positives, but at the expense of including more false positives. One still sees, however, that the observations from the other approximation still hold, namely, that volumes of representation are both redundant and separate from one another.(TIF)

Figure S2 **Volumes of representation of a nonlinear function over input sequences.** Approximation of order-3 volumes of representation of the task Parity-3 binary input to a SIP-RN. By an appropriate union of these volumes, the volumes of representation of the outcome 0 (green) and 1 (orange) are identified. The approximation uses the mean and standard deviation of the coordinates. While the first three principal components are sufficient for showing distinct order-3 volumes of representation, more dimensions are necessary to illustrate separate volumes of the outcome of the nonlinear function. The separability of the function's outcomes explains the ability of optimal linear classifiers to successfully perform the nonlinear task.
(TIF)

Figure S3
**Average classification performance using the Hamming distance of the network states from the vertexes of autonomous attractors.** 100 networks are trained by STDP and IP simultaneously on (A) the memory task RAND x 4, (B) the prediction task Markov-85, and (C) the nonlinear task Parity-3. Given the input set 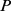, and the family of discrete-time autonomous semi-dynamical systems generating these networks 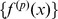, the network states comprising the autonomous attractor (the attractor's vertexes) are identified as follows. First, initial conditions are selected within the input-sensitive basin of attraction. Second, the input is clamped to one member of 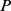. Third, the solution of 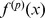 is generated for a sufficient number of time steps, so that the dynamics, following a transient period, converges to the attractor. Training and testing optimal linear classifiers is carried through as before. The training and testing data is, however, the Hamming distance between the network states and the vertexes of the attractors. Error bars indicate standard error of the mean. The red line marks chance level. The *x*-axis shows the input time-lag. Negative time-lags indicate the past, and positive ones, the future.(TIF)

Figure S4 **Average classification performance of networks combining the weights of SP-RNs and thresholds of IP-RNs.** 100 networks are trained by STDP and IP simultaneously (orange), IP alone (blue), or trained by STDP alone followed by injecting the thresholds resulting from IP at the end of the plasticity phase (green) on (A) the memory task RAND x 4, (B) the prediction task Markov-85, and (C) the nonlinear task Parity-3. The combined networks (green) lack the contribution of the interaction between synaptic and intrinsic plasticity during the plasticity phase. This results in their performance being inferior to the networks where synaptic and intrinsic plasticity interact. Error bars indicate standard error of the mean. The red line marks chance level. The *x*-axis shows the input time-lag. Negative time-lags indicate the past, and positive ones, the future.
(TIF)

Text S1 **Comparing nonplastic networks.**
(PDF)

Text S2 **Long-term behavior of learning.**
(PDF)

Text S3 **Definitions of nonautonomous dynamical systems.**
(PDF)
