# Supplementary figures and images for: Spatiotemporal Computations of an Excitable and Plastic Brain: Neuronal Plasticity Leads to Noise-Robust and Noise-Constructive Computations
Source: PLoS Comput Biol. 2014 Mar 20;10(3):e1003512. doi: 10.1371/journal.pcbi.1003512 (PMC3961183; doi:10.1371/journal.pcbi.1003512)

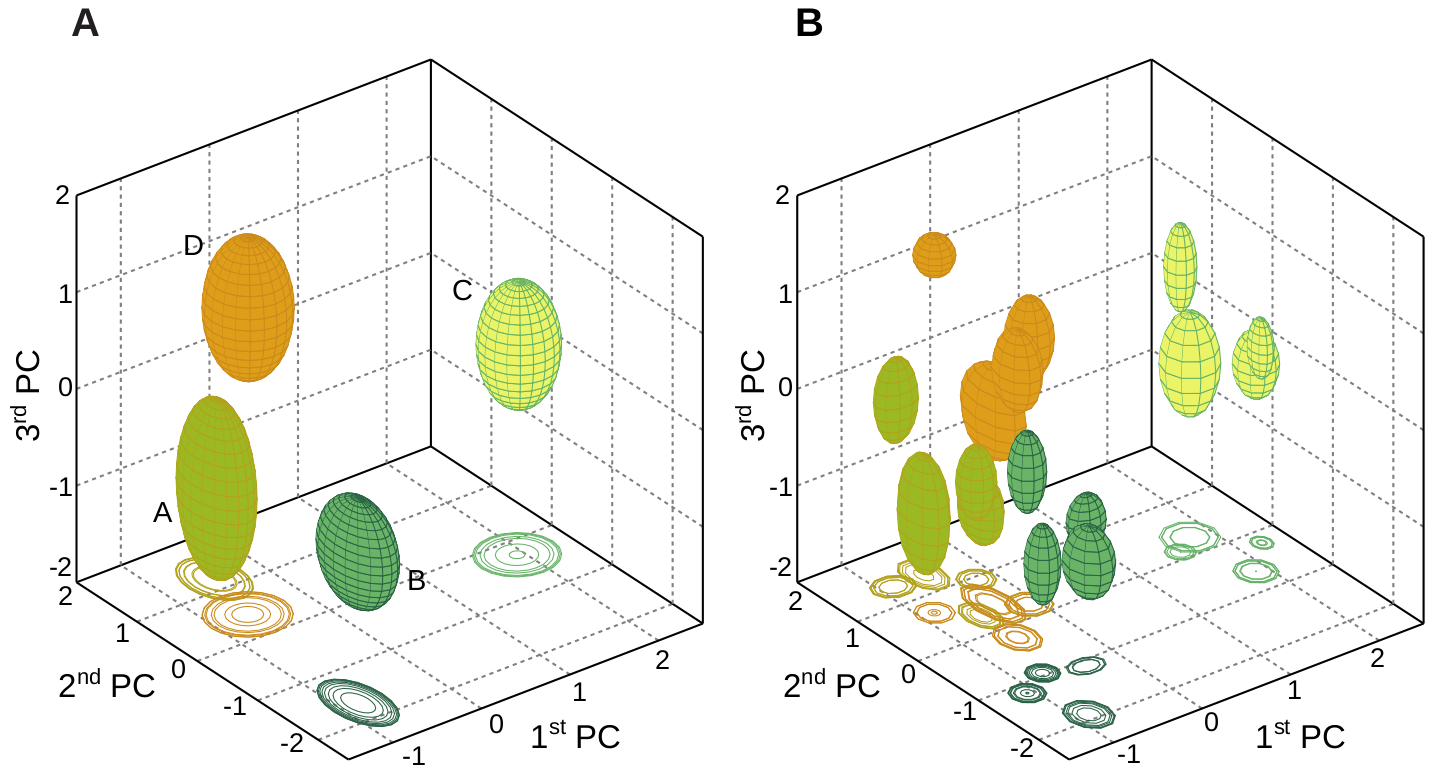

Supplement: Figure S1 — Approximating volumes of representation using percentiles. (A) Percentile approximation of the order-1 volumes of representation of a SIP-RN. (B) Percentile approximation of the order-2 volumes of representation of a SIP-RN. Order-2 volumes are more exact approximations to the order-1 volumes according to the volumes' inclusion property. The correspondence is clarified by using similar color coding. (A,B) This approximation is done as follows. After transforming the network states to the principal components space, the coordinates of the first three principal components belonging to each volume of representation are first bootstrapped to 10000 samples, and the and percentiles are computed. Each volume is then approximated by an ellipsoid whose semi-axes extend to these percentiles and is centered at their average. This alternative approximation is less liberal than the one that uses means and standard deviations in that it extends the ellipsoids to assure including more true positives, but at the expense of including more false positives. One still sees, however, that the observations from the other approximation still hold, namely, that volumes of representation are both redundant and separate from one another. (TIF) [file pcbi.1003512.s001.tif]

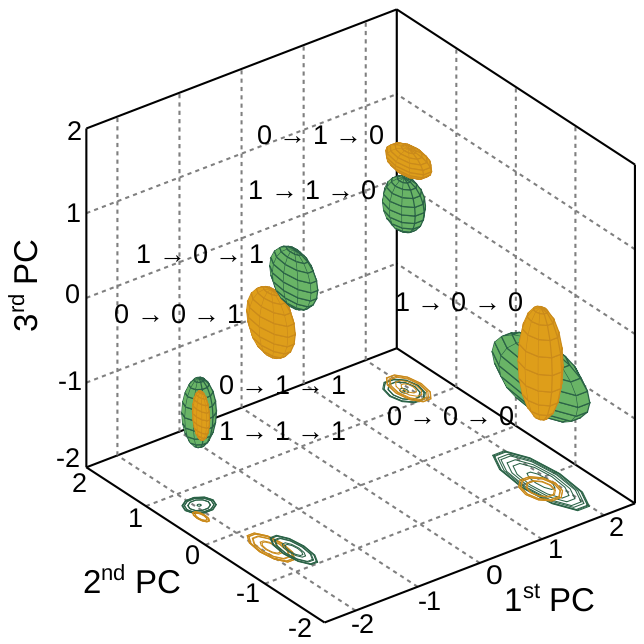

Supplement: Figure S2 — Volumes of representation of a nonlinear function over input sequences. Approximation of order-3 volumes of representation of the task Parity-3 binary input to a SIP-RN. By an appropriate union of these volumes, the volumes of representation of the outcome 0 (green) and 1 (orange) are identified. The approximation uses the mean and standard deviation of the coordinates. While the first three principal components are sufficient for showing distinct order-3 volumes of representation, more dimensions are necessary to illustrate separate volumes of the outcome of the nonlinear function. The separability of the function's outcomes explains the ability of optimal linear classifiers to successfully perform the nonlinear task. (TIF) [file pcbi.1003512.s002.tif]

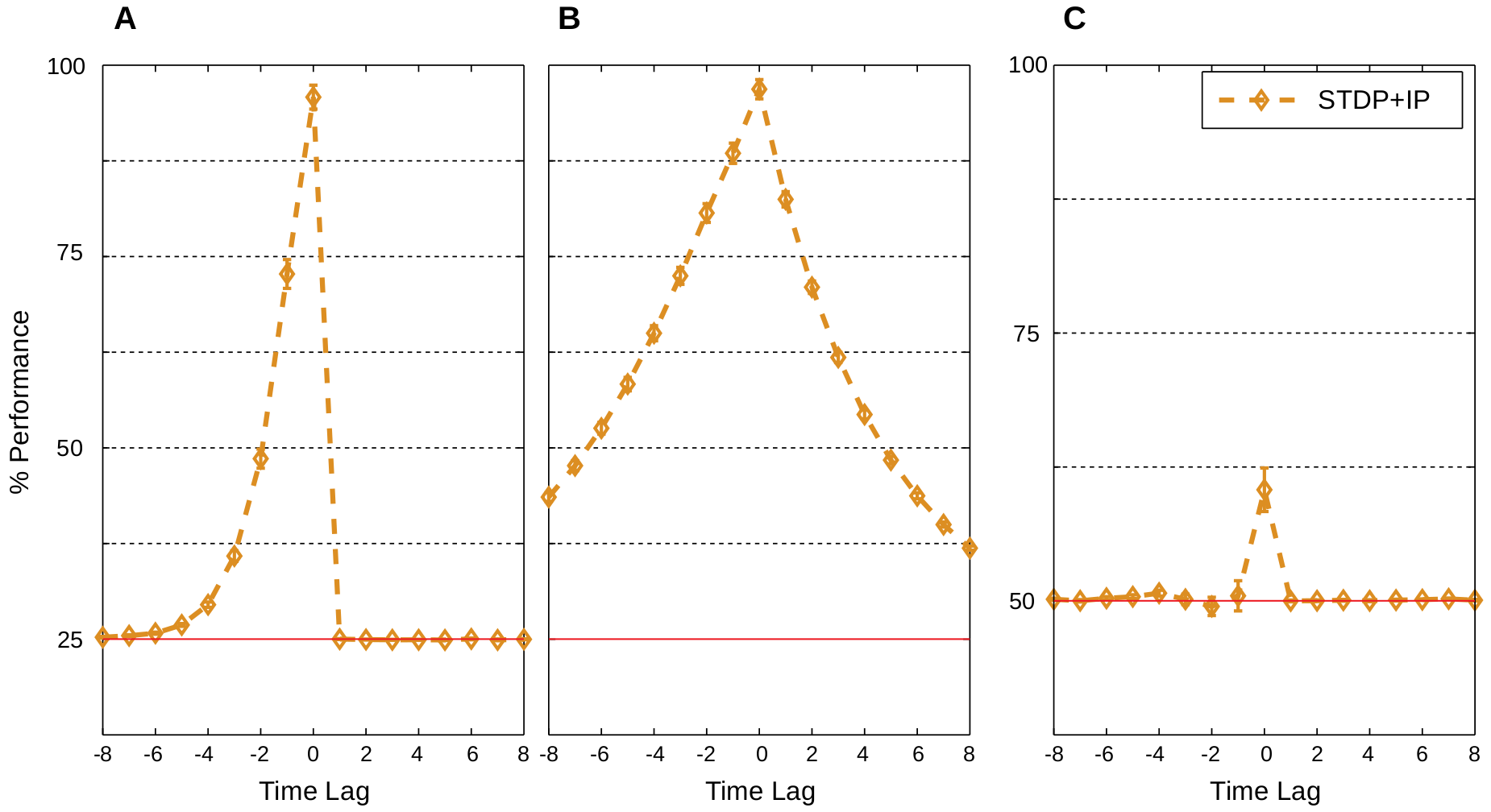

Supplement: Figure S3 — Average classification performance using the Hamming distance of the network states from the vertexes of autonomous attractors. 100 networks are trained by STDP and IP simultaneously on (A) the memory task RAND x 4, (B) the prediction task Markov-85, and (C) the nonlinear task Parity-3. Given the input set , and the family of discrete-time autonomous semi-dynamical systems generating these networks , the network states comprising the autonomous attractor (the attractor's vertexes) are identified as follows. First, initial conditions are selected within the input-sensitive basin of attraction. Second, the input is clamped to one member of . Third, the solution of is generated for a sufficient number of time steps, so that the dynamics, following a transient period, converges to the attractor. Training and testing optimal linear classifiers is carried through as before. The training and testing data is, however, the Hamming distance between the network states and the vertexes of the attractors. Error bars indicate standard error of the mean. The red line marks chance level. The x-axis shows the input time-lag. Negative time-lags indicate the past, and positive ones, the future. (TIF) [file pcbi.1003512.s003.tif]

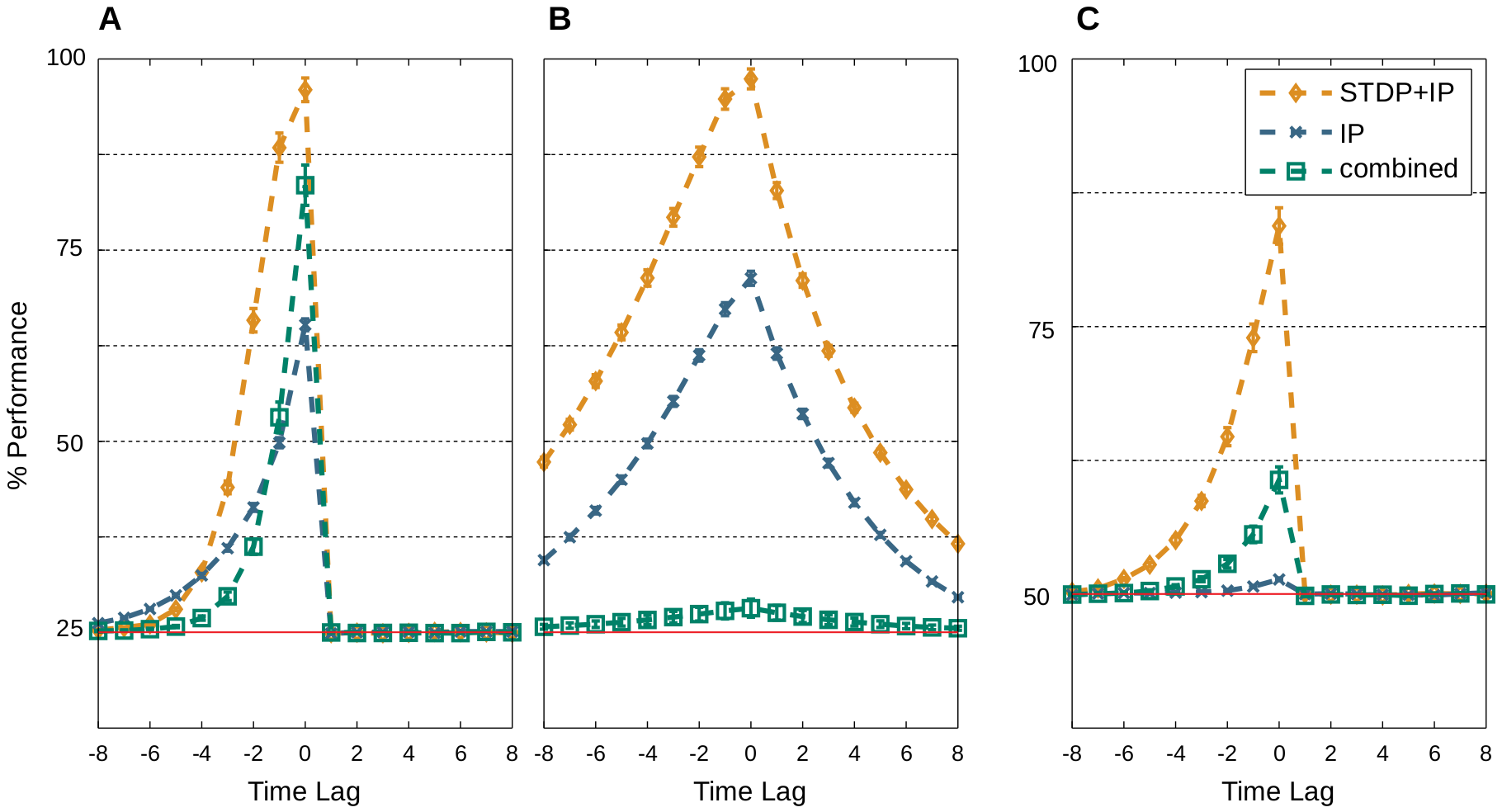

Supplement: Figure S4 — Average classification performance of networks combining the weights of SP-RNs and thresholds of IP-RNs. 100 networks are trained by STDP and IP simultaneously (orange), IP alone (blue), or trained by STDP alone followed by injecting the thresholds resulting from IP at the end of the plasticity phase (green) on (A) the memory task RAND x 4, (B) the prediction task Markov-85, and (C) the nonlinear task Parity-3. The combined networks (green) lack the contribution of the interaction between synaptic and intrinsic plasticity during the plasticity phase. This results in their performance being inferior to the networks where synaptic and intrinsic plasticity interact. Error bars indicate standard error of the mean. The red line marks chance level. The x-axis shows the input time-lag. Negative time-lags indicate the past, and positive ones, the future. (TIF) [file pcbi.1003512.s004.tif]
