# Supplementary material for: Spatiotemporal Computations of an Excitable and Plastic Brain: Neuronal Plasticity Leads to Noise-Robust and Noise-Constructive Computations
Source: PLoS Comput Biol. 2014 Mar 20;10(3):e1003512. doi: 10.1371/journal.pcbi.1003512 (PMC3961183; doi:10.1371/journal.pcbi.1003512)
Supplement: Text S1 — Comparing nonplastic networks. (PDF) [file pcbi.1003512.s005.pdf]

S1. Comparing nonplastic networks

Comparing SIP-RNs and nonplastic networks requires generating random networks with reasonable weight and threshold distributions. Usually, the spectral radius of the weight matrix is scaled "appropriately" in the reservoir computing community, such that the recurrent network achieves the echo state property, which assures a decaying memory of the network's input. Unfortunately, no heuristics from the reservoir computing literature exist for kWTA networks. Using the spectral radius scaling as a rule of thumb is no guarantee to make the network better or worse, since it has no theoretical grounding of its effect without taking the model neural network into account, along with its input statistics. Sufficient conditions for the existence of the echo-state property, and that relates its existence with certain input statistics and spectral properties of the weight matrix, is only formally stated for hyperbolic tangent sigmoid neurons, as is proven in [1]. Given the above, we see no existing heuristics in the literature for selecting weights and thresholds of our kWTA networks and our given input distributions, and using any would jeopardize the formality of our treatment.

For a fair comparison between SIP-RNs and nonplastic networks, we devised a procedure for generating random networks that have the same weight and firing threshold distributions as plastic networks. In a first set of networks, weights were trained with STDP and then shuffled to assure the destruction of structure, while keeping the weight distribution comparable to SP-RNs. These networks showed significantly lower average performance than SIP-RNs in all tasks, as is demonstrated in Figure 1.

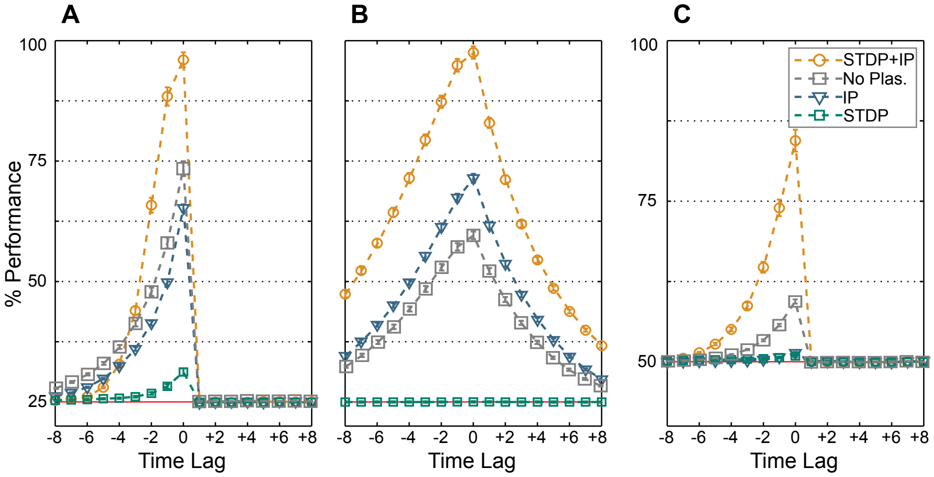

Figure 1. Average classification performance. 100 networks are trained by STDP and IP simultaneously (orange), IP alone (blue), STDP alone (green), or they are nonplastic (gray). Optimal linear classifiers are then trained to perform (A) the memory task RAND×4, (B) the prediction task Markov-85, and (C) the nonlinear task Parity-3. Nonplastic networks have their weights trained by STDP and then randomly shuffled, so that they have the same weight and threshold distributions as SP-RNs. However, due to the shuffling, their weight matrices carry no structure. Error bars indicate standard error of the mean. The red line marks chance level. The x-axis shows the input time-lag. Negative time-lags indicate the past, and positive ones, the future.

In order to show that the high performance of SIP-RNs is a result of the structure imposed by synaptic and intrinsic plasticity on the network' connectivity and the neurons' firing thresholds, we trained a second set of networks on both plasticity mechanisms. We then randomly shuffled both their weights and thresholds so that they have the same weight and threshold distributions as SIP-RNs. The performance

of these networks, compared to networks where only the weights were trained and shuffled, depends on the task. They perform better on the prediction task, but worse on the memory and nonlinear tasks (see Figure 2). Nevertheless, they still show significantly lower average performance than SIP-RNs.

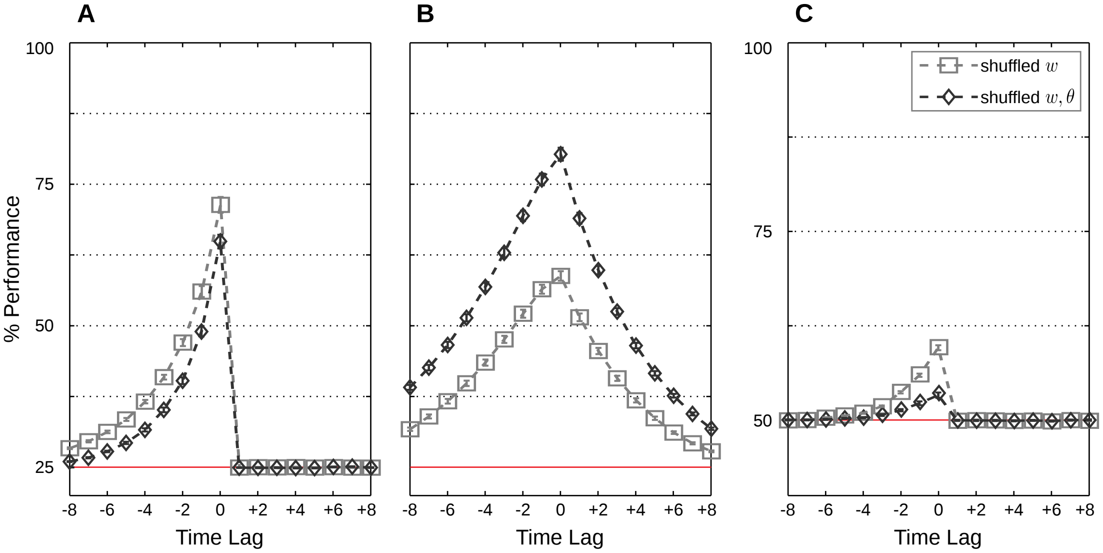

Figure 2. Performance of random networks. 100 networks are either trained by STDP alone or by both STDP and IP.The weights w in the first group were shuffled (gray), while both the weights w and the thresholds θ were shuffled in the second group (black). Optimal linear classifiers are then trained to perform (A) the memory task RAND×4, (B) the prediction task Markov-85, and (C) the nonlinear task Parity-3. Error bars indicate standard error of the mean. The red line marks chance level. The x-axis shows the input time-lag. Negative time-lags indicate the past, and positive ones, the future.

It might still be possible to find, in random networks, ones that are comparable or ones that outperform our best SIP-RNs, but we suppose that it is expensive for the genetic code to determine these networks, and therefore brain plasticity was the solution for this limitation. These hypothetical networks might also not have properties of the input statistics embedded in their structure and spontaneous behavior, as our plastic networks show. They also might not be generically better for all stimuli, while our networks adapt to their stimuli.

References

1. Manjunath G, Jaeger H (2013) Echo state property linked to an input: Exploring a fundamental characteristic of recurrent neural networks. Neural Comput 25: 671–696.
