# Supplementary material for: Spatiotemporal Computations of an Excitable and Plastic Brain: Neuronal Plasticity Leads to Noise-Robust and Noise-Constructive Computations
Source: PLoS Comput Biol. 2014 Mar 20;10(3):e1003512. doi: 10.1371/journal.pcbi.1003512 (PMC3961183; doi:10.1371/journal.pcbi.1003512)
Supplement: Text S2 — Long-term behavior of learning. (PDF) [file pcbi.1003512.s006.pdf]

S2. Long-term behavior of learning

A limitation of the current model is the nonconvergence of learning. As Fig. 3 shows, the `SIP-RN`s mutual information with their input degrades for longer plasticity exposure, which results in a decrease in their computational performance. This degradation is due to the unoptimized time scales at which synaptic and intrinsic plasticity are operating. At some point, intrinsic plasticity becomes unable to keep up with synaptic plasticity that reinforces the input-insensitive dynamics. A possible solution would be to implement some form of *metaplasticity* [1] that operates on the learning rates of the plasticity mechanisms, increasing or decreasing their effects, as necessary, or to introduce synaptic scaling as an additional mechanism that prevents the input-insensitive regime from dominating. However, either solution would make the model more complicated than intended for this work, where we aimed at providing, for the first time, a nonautonomous dynamical systems treatment on the roles and interaction of `STDP` and `IP` for spatiotemporal computations in recurrent neural networks.

References

1. Abraham WC, Philpot B (2009) Metaplasticity. Scholarpedia 4: 4894.
