# Supplementary material for: Spatiotemporal Computations of an Excitable and Plastic Brain: Neuronal Plasticity Leads to Noise-Robust and Noise-Constructive Computations
Source: PLoS Comput Biol. 2014 Mar 20;10(3):e1003512. doi: 10.1371/journal.pcbi.1003512 (PMC3961183; doi:10.1371/journal.pcbi.1003512)
Supplement: Text S3 — Definitions of nonautonomous dynamical systems. (PDF) [file pcbi.1003512.s007.pdf]

S7. Definitions of nonautonomous dynamical systems

The following definitions are adapted from [1, 2].

The *process* definition of a discrete-time nonautonomous dynamical system can be stated as follows:

Definition 1. Let (X, d) be a metric space with a metric d. A *discrete-time process* is a function $\phi : \mathbb{Z}^2_{\geq} \times X \to X$ that satisfies

1. $\phi(t_0, t_0, x) = x \quad \forall t_0 \in \mathbb{Z}$ and $\forall x \in X$.
2. $\phi(t_2, t_0, x) = \phi\big(t_2, t_1, \phi(t_1, t_0, x)\big) \quad \forall t_0 \leq t_1 \leq t_2 \in \mathbb{Z}$ and $\forall x \in X$.
3. ϕ is continuous.

One may reformulate a discrete-time process as a discrete-time autonomous semi-dynamical system on the extended state space $\mathcal{X} := \mathbb{Z} \times X$ with the mapping

$$\zeta\big(t, (t_0, x_0)\big) := \big(t + t_0, \phi(t + t_0, t_0, x_0)\big) \text{ for all } \big(t, (t_0, x_0)\big) \in \mathbb{Z}^+_0 \times \mathcal{X}$$

However, no compact set in $\mathcal{X}$ is invariant under ζ, which necessitates extending the concepts of invariance and attractivity in ϕ, as we show in the main text.

An alternative formulation of nonautonomous dynamical systems is given by a *skew product flow*

Definition 2. Let (X, d_X) and (P, d_P) be metric spaces with metrics d_X and d_P. A *discrete-time nonautonomous dynamical system* (ψ, φ) is defined as follows:

- a *discrete-time dynamical system* ψ on a parameter space P is a group of homeomorphisms $(\psi_t)_{t\in\mathbb{Z}}$ under composition on P such that
  1. $\psi_0(p) = p$ for all $p \in P$.
  2. $\psi_{t_1+t_2} = \psi_{t_2}\big(\psi_{t_1}(p)\big)$ for all $t_1, t_2 \in \mathbb{Z}$.
  3. the mapping $(t, p) \mapsto \psi_t(p)$ is continuous.
- and a *cocycle mapping* $\varphi : \mathbb{Z}^+_0 \times P \times X \to X$ that satisfies
  1. $\varphi(0, p, x) = x$ for all $(p, x) \in P \times X$.
  2. $\varphi(t_1 + t_2, p, x) = \varphi\big(t_2, \psi_{t_1}(p), \varphi(t_1, p, x)\big)$ for all $t_1, t_2 \in \mathbb{Z}^+_0, (p, x) \in P \times X$.
  3. the mapping $(t, p, x) \mapsto \varphi(t, p, x)$ is continuous.

As with the process formulation, one may define a discrete-time autonomous semi-dynamical system $\zeta : \mathbb{Z}^+_0 \times \mathcal{X} \to \mathcal{X}$ on the extended state space $\mathcal{X} := P \times X$. ζ is called the *skew product flow* associated with the nonautonomous semi-dynamical system (ψ, φ) on $\mathcal{X}$, and is given by

$$\zeta\big(t, (p, x)\big) := \zeta\big(\theta_t(p), \varphi(t, p, x)\big)$$

The skew product flow of the systems we considered here is more specific, since it falls under the class of *random dynamical systems* [3], the treatment of which at this point would only serve in complicating the analysis. However, with its general form presented above, the skew product formulation is more intuitive than the process formulation regarding input-driven dynamical systems, since the input dynamics in the former is explicitly stated by the driving dynamical system ψ, and is only implicitly considered in the case of a process. On the other hand, the process formulation is simpler and is sufficient for drawing the results and conclusions of the main text.

References

1. Kloeden PE, Rasmussen M (2011) Nonautonomous dynamical systems. AMS Bookstore.

2. Kloeden PE, Pötzsche C, Rasmussen M (2013) Discrete-time nonautonomous dynamical systems. In: Johnson R, Pera M, editors, Stability and Bifurcation Theory for Non-Autonomous Differential Equations, Springer. pp. 35–102.

3. Arnold L (1998) Random dynamical systems. Springer Berlin.
